# Transcription Factors in *Escherichia coli* Prefer the *Holo* Conformation

**DOI:** 10.1371/journal.pone.0065723

**Published:** 2013-06-12

**Authors:** Yalbi Itzel Balderas-Martínez, Michael Savageau, Heladia Salgado, Ernesto Pérez-Rueda, Enrique Morett, Julio Collado-Vides

**Affiliations:** 1 Programa de Genómica Computacional, Centro de Ciencias Genómicas, Universidad Nacional Autónoma de México, Cuernavaca, Morelos, México; 2 Department of Biomedical Engineering, University of California Davis, Davis, California, United States of America; 3 Departamento de Ingeniería Celular y Biocatálisis, Instituto de Biotecnología, Universidad Nacional Autónoma de México, Cuernavaca, Morelos, México; Baylor College of Medicine, United States of America

## Abstract

The transcriptional regulatory network of *Escherichia coli* K-12 is among the best studied gene networks of any living cell. Transcription factors bind to DNA either with their effector bound (*holo* conformation), or as a free protein (*apo* conformation) regulating transcription initiation. By using RegulonDB, the functional conformations (*holo* or *apo*) of transcription factors, and their mode of regulation (activator, repressor, or dual) were exhaustively analyzed. We report a striking discovery in the architecture of the regulatory network, finding a strong under-representation of the *apo* conformation (without allosteric metabolite) of transcription factors when binding to their DNA sites to activate transcription. This observation is supported at the level of individual regulatory interactions on promoters, even if we exclude the promoters regulated by global transcription factors, where three-quarters of the known promoters are regulated by a transcription factor in *holo* conformation. This genome-scale analysis enables us to ask what are the implications of these observations for the physiology and for our understanding of the ecology of *E. coli*. We discuss these ideas within the framework of the demand theory of gene regulation.

## Introduction

Transcription factors (TFs) are usually allosteric proteins that bind specifically to their operator DNA sites, which are usually located near promoters, either in the *holo* or *apo* conformation to regulate gene expression. We refer to a functional *holo* conformation when the TF binds to DNA as a complex bound to an effector that can be either a noncovalently bound small molecule, or after a covalent modification, such as phosphorylation by a two-component system; whereas a TF binds in an *apo* conformation when the protein binds alone. For instance, CRP binds to its specific binding sites once bound to cAMP, its allosteric small ligand; whereas the LacI repressor binds to DNA as a protein in *apo* conformation, and unbinds in the presence of allolactose, its allosteric modifier.

The best-described transcriptional regulatory network (TRN) of any living organism, that of *Escherichia coli* K-12, provides a detailed description of TFs, including the characterization of their mode of control (activator, repressor, or dual) and the functional conformation that binds to DNA operator sites. The wealth of knowledge available in RegulonDB [1] includes as well the classification of TFs into their corresponding evolutionary families, the specific promoters subject to regulation of these TFs, and the functional classification of the regulated genes. Taking all these pieces of knowledge together, enabled us in this paper to address questions concerning relationships of the mode of control, and the conformation of TFs in relation to the functional classes and the evolutionary families of TFs. Briefly, we performed a genome-wide analysis of the relationship between physiology and mechanisms of gene regulation for this bacterium.

Extensive molecular studies in *E. coli* K-12 have characterized the regulatory role and the effector associated with the functional conformation of each TF. This information has allowed a comprehensive global understanding of gene regulation that cannot be obtained through the study of individual genetic systems. For instance, the functional conformation for modeling regulatory networks in order to elucidate the design principles for transcriptional regulation of bacterial TFs in elementary gene circuits, have previously been incorporated [2]; however these principles are still the focus of further research in this area [3].

In this work we address the question of whether a preferential functional conformation is used by TFs in *E. coli*, and what are the biological implications. We found a high preference of activators and repressors for the *holo* functional conformation whereas, interestingly, activators very seldom have an *apo* functional conformation. Given that many TFs have dual repressor/activator functions, we also analyzed the conformational distribution of individual TF-promoter pairs, since in most cases TFs work either activating or repressing specific promoters. At this level of individual interactions, we found the same asymmetric distribution, with a preference for the *holo* functional conformation. In conclusion, the major properties of the TRN we found are: (a) extremely rare *apo* activation both at the level of TFs (only one TF activates its promoters in *apo* conformation) and at the level of individual TF-promoter pairs, together with (b) a high tendency for promoters to be regulated at least by one TF in a *holo* conformation and (c) a high frequency of dual regulation. We analyzed both functional and evolutionary hypotheses in searching for an explanation for these striking observations, and we discuss our results within the framework of the demand theory of gene regulation [4], [5].

## Results

### Classification of TFs Based on their Functional Conformation

RegulonDB version 7.0 describes experimental evidence for a total of 149 TFs governing 732 promoters [1]. From this set, 98 TFs (66%) have an associated effector that can be either an allosteric, noncovalently bound, small molecule (70 TFs) or a covalent modification (phosphorylation) by a two-component system (28 TFs). The 98 TFs with effectors were classified both by their mode of control (activator, repressor, or dual) and by their functional conformation (*holo*, *apo*, or *holo-apo*). We will only refer to the *functional conformation* throughout the text (Figure S1 and Tables S1 and S2).

### Apo/holo Asymmetry at the Level of TFs

**Figure 1:**
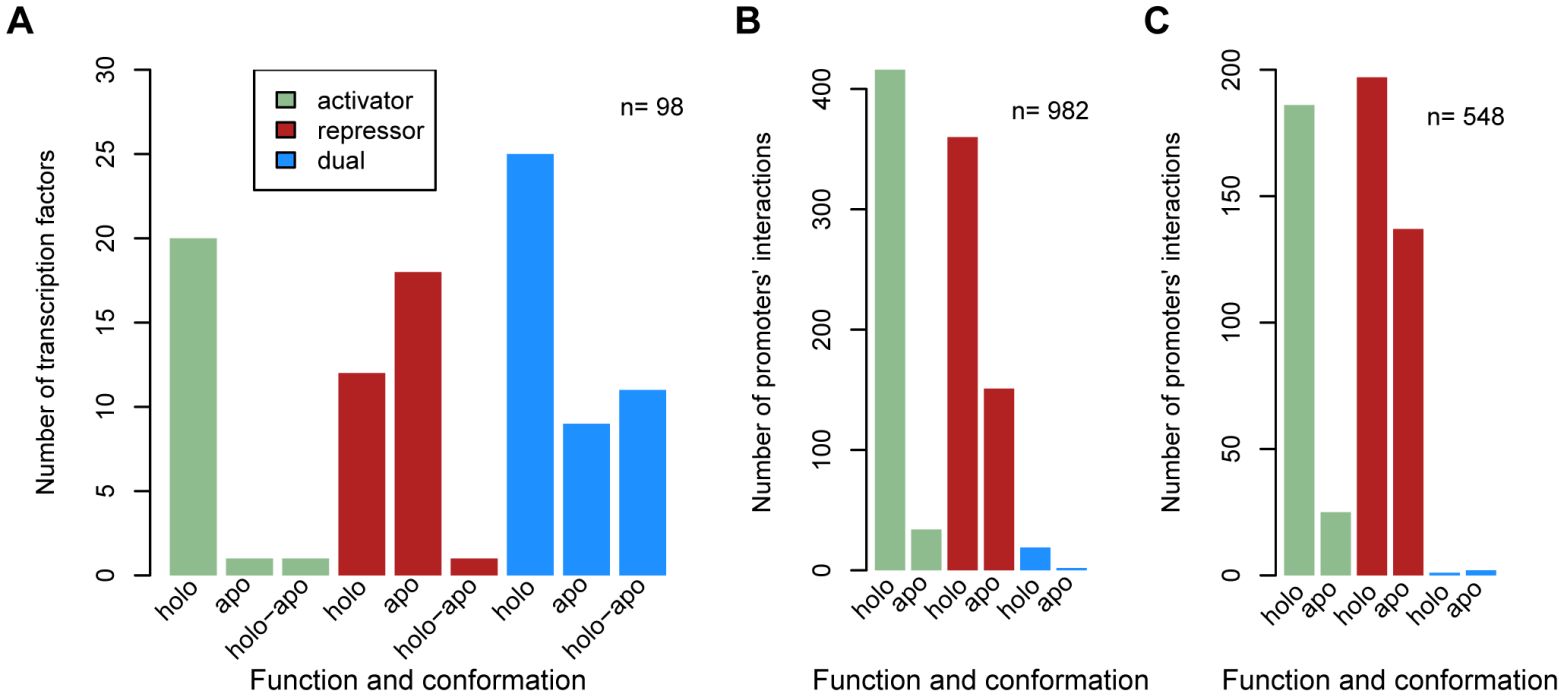
Conformational asymmetries. (a) Conformational asymmetries of TFs. TFs were classified based on the mode of control (activators: green; repressors: red; dual regulators: blue) and the functional conformation (holo, apo, or holo-apo). Pearson’s chi-squared test: χ^2^ = 29.0212, df = 4, P = 7.74×10^−06^. (b) Conformational asymmetries in TF functional conformation-promoter pairs. TF functional conformation-promoter pairs were classified according to the mode of control (activation: green, repression: red, dual: blue) and the functional conformation (holo, apo, or holo-apo) of the TF. Activating interaction pairs may come from TFs that are either activators or from promoters that are activated by dual TFs; repressing interaction pairs may come from repressor TFs or from promoters negatively regulated by dual TFs. Dual interaction pairs refer here exclusively to interactions by a TF with a dual effect on the same promoter. Pearson’s chi-squared test: χ^2^ = 76.3451, df = 2, P<2.2×10^−16^. (c) Effect of excluding global TFs on conformational asymmetries of TF functional conformation-promoter pairs. Only TF-promoter interactions where the TF is local are here counted. If a promoter is subject both to local and global regulation, those interactions with local TFs contribute to this counting. Interactions, excluding those by global regulators. They were classified according to the mode of control (activation: green; repression: red; dual: blue) and functional conformation (holo, apo, or holo-apo) of the TF. Pearson’s chi-squared test: χ^2^ = 79.4576, df = 4, P = 2.269×10^−16^.

As shown in Figures 1a and S1, a striking discovery was that 95% of activators regulate in the *holo* conformation, with the exception of Cbl, which regulates in *apo* conformation (5%) and is required for the expression of sulfate starvation genes [6]. On the contrary, functional repressors bind either in the *apo* (60%) or in the *holo* (40%) conformation. For dual regulators, 55.5% have the *holo* conformation, 20% the *apo* conformation, and 24.5% the *holo-apo* conformation (which like Lrp bind functionally both in *apo* and in *holo*) [7]. A chi-square test showed a significant correlation between the functional conformation and the TF’s mode of control, with a *P* value of 7.74×10^−6^. In summary, activators and dual TFs bind their target sites predominantly in the *holo* conformation.

### Conformation is not Conserved within Evolutionary Families

In order to evaluate whether the conformational distribution is a result of the evolutionary history of TFs, we analyzed TF families with at least five or more members. We found that the functional conformation was not conserved within evolutionary TF families as shown in Figure S2. A clear exception was the family of two-component systems, which are those with a histidine protein kinase and a response TF protein. TFs are active when phosphorylated, thus all of them are functional in the *holo* conformation, except for TorR which is functional in *apo* and *holo* [8]. They can be either activators or dual regulators. Indeed, the diverse allosteric mechanisms within homologous proteins have been suggested to result from the evolution of allosteric interactions within colocalized molecules [9]–[11].

### Apo/holo Asymmetry at the Level of TF-promoter Pairs

The mode of control (activation or repression), is more precisely defined at the level of TFs governing individual promoters. We defined TF functional conformation-promoter pairs (TF-promoter pairs) irrespective of the number of TF-binding sites with the same effect on a promoter. When a promoter was controlled by more than one TF, each pair was counted separately. Of 1,327 TF functional conformation-promoter pairs counted, 982 were among TFs with a known effector and functional conformation Out of these 982 TF-promoter pairs, we find the following distribution: *holo* repression (37%), *holo* activation (42%), *apo* repression (15%), *apo* activation (3.25%, with 2 contributed by only 1 TF activator, Cbl, and 32 by 12 dual TFs), dual regulation in *holo* conformation (1.93%) and dual regulation in *apo* conformation (0.2%). Dual TFs accounted for 372 *holo* activation and 293 *holo* repression pairs. Only a few promoters were subject to dual regulation by the same TF (19 promoters in *holo* and 2 in *apo* conformation). We observed a significant correlation between the mode of control and functional conformation (*P*<2.2×10^−16^) (Figures 1b and S3). Furthermore, we also analyzed TFs that had many sites in the same promoter region, and we found a significant correlation with the *holo* conformation (Figure S4). Briefly, at the level of TF-promoter pairs, regulation in the *holo* conformation is clearly dominant.

### Global TFs Act in the Holo Conformation

We analyzed global TFs, which have many interactions and satisfy additional criteria as defined in methods, to see if their inclusion might modify the distributions of *holo* and *apo* conformations. Six global TFs that have an associated effector were considered (with the number of promoters they regulate shown in parentheses): CRP (210), FNR (76), ArcA (48), Fur (36), Lrp (34), and NarL (30). These account for 44.1% of the interaction pairs with a known effector. Most of the global TFs with an effector have a functional *holo* conformation; Lrp is an exception, as it regulates in both the *holo* and *apo* conformations [12]. None of the global TFs regulate exclusively in the *apo* conformation. As shown in Figures 1c and S5, global TFs use the *holo* conformation for both activation and repression, contributing significantly to the total number of *holo* repressor interaction pairs; however, even if we excluded all pairs contributed by global TFs, *apo* activators were still underrepresented, accounting for only 34 out of 548 interactions, as shown in Fig. 1c. We also did the same analysis excluding the promoters regulated by two-component systems and, although the bias is almost lost, we still find few promoters that are *apo* activated (Figure S6).

### Distribution of TFs Functional Conformations in Complex Regulation

**Figure 2:**
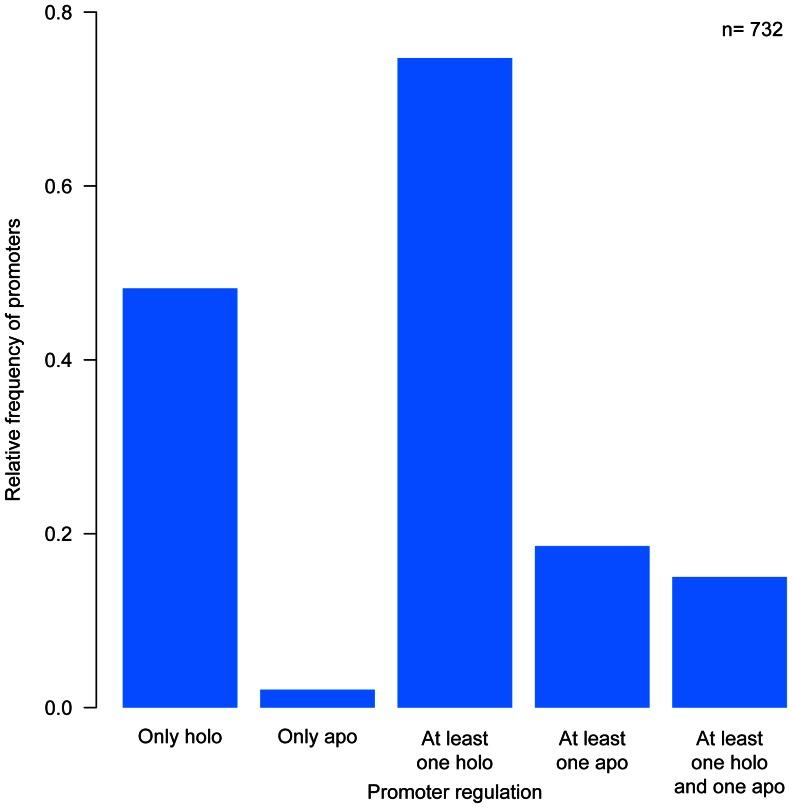
Complex regulation and TF conformational tendency. With this classification system, we considered promoters to be regulated by one or more TFs, but each of them with the same mode and conformation. Each bar corresponds to the relative frequency of a promoter regulated by at least one TF only in holo functional conformation, only in apo functional conformation, at least one TF in holo, at least one TF in apo conformation, or at least one TF in holo and one in apo conformation. The colors inside each bar correspond to the contributions by promoters subject to only one TF, two, three or more.

Simple and complex regulation are usually defined in terms of how many TFs affect a promoter [13]. Our data offers a different perspective on the combination of TF-promoter pairs by taking into consideration the functional conformation of the TF. Thus, complex conformational regulation involves different conformations and modes of control (activation or repression) of TFs, irrespective of the number of TFs and of binding sites for each TF (see Methods). For instance, *malE* is subject to simple conformational regulation, even though it is controlled by two activators (CRP and MalT) in the *holo* conformation. On the contrary, *lacZYA* is subject to complex regulation, because it is controlled by one repressor in *apo* conformation and one activator in *holo* conformation (LacI and CRP, respectively). By this criterion, 44% of all 732 promoters are subject to complex conformational regulation, whereas the other 56% involve simple conformational regulation (Figures S7, S8, S9 and S10). As shown in Figures 2, and S11, 75% of all promoters are regulated by at least one TF in *holo* conformation. Although 22% are regulated by at least one TF in *apo* conformation, most of them also include a TF in the *holo* conformation. The high occurrence of *holo* conformation reflects the observation that global regulators, which work essentially in *holo* conformation, are usually participating in both simple and complex conformational regulation.

### Physiological Implications of Holo and Apo Conformations

**Figure 3:**
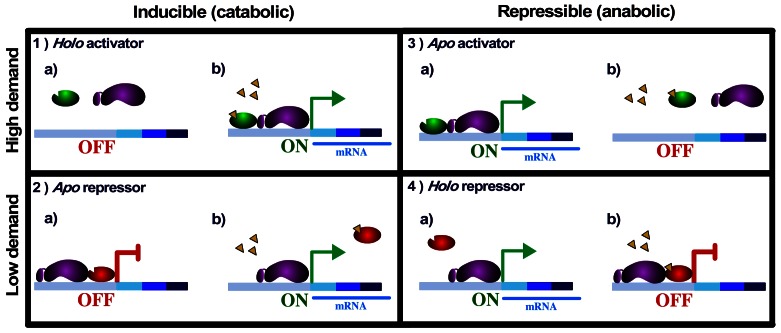
Predicted gene control circuits for simple cases. **Case 1.** Inducible catabolic high-demand system. a) Expression of the regulated genes is OFF, because the activator is in a nonfunctional state. b) In the presence of the effector, it binds to the activator, changing it to the holo conformation, which facilitates transcription, e.g., maltotriose binds to MalT and this induces maltose operon expression. Case 2. Inducible catabolic low-demand system. a) The repressor is functional in the apo conformation, so the system is repressed in the absence of the effector. b) In the presence of the effector, it binds to the TF, changing it to a nonfunctional conformation, which allows induction of the system, e.g., allolactose binds to LacI and this induces lactose operon expression. Case 3. Repressible anabolic high-demand system. a) In the absence of the effector, the system is ON, with the activator in the apo conformation. b) When the effector is present, the activator is nonfunctional and the system is deactivated, e.g., Cbl activates the tau and ssi operons when it is unbound to the adenosyl 5′-phosphosulfate compound. Case 4. Repressible anabolic low-demand system. a) The repressor is nonfunctional in the absence of effector, so gene expression is turned ON. b) In the presence of effector, it binds to the TF, converting it to the holo conformation, which binds DNA and represses transcription, e.g., TrpR bound to tryptophan in the holo conformation represses the tryptophan operon. Symbols: ON and OFF show gene expression and a lack of gene expression, respectively. Activator: green oval; repressor: red oval; RNA polymerase: purple bean shape; effector: yellow triangles; mRNA: blue line.

**Figure 4:**
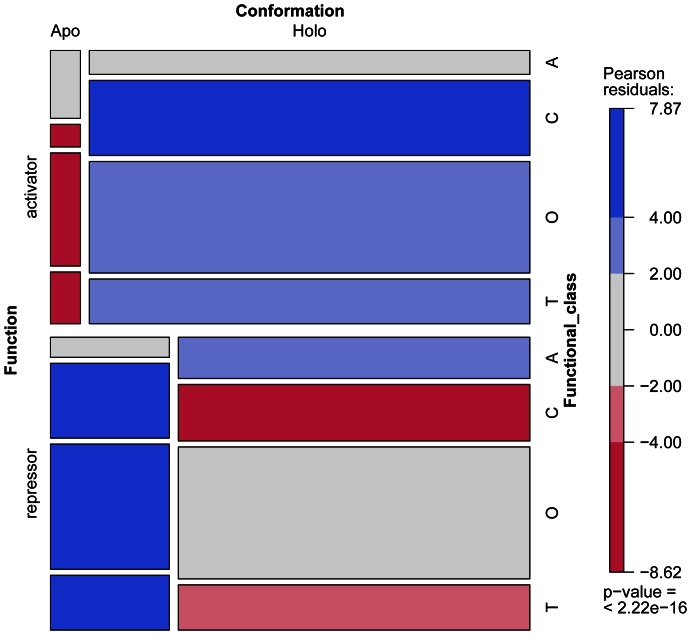
Gene classification based on MultiFun and TF conformational bias. Each gene was classified by its regulation, on the function of the TF (activator or repressor), the conformation (apo or holo), and functional class (T: transport; O: others; C: catabolic; A: anabolic). This plot represents a contingency table, with each rectangle corresponding to a piece of the plot, with their sizes proportional to the cell entry. The Pearson residuals indicate the fit of a log-linear model. Blue represents the maximum significance of the corresponding residual, and red shows the minimum.

When focusing on the change in expression levels of target genes, systems have been classified as inducible or repressible. Thus, at least four types of gene control circuits can be defined in this context: induction with activator or repressor control, and repression with activator or repressor control [3], [14] (See Figure 3). It has been observed that catabolic systems tend to be inducible, whereas anabolic systems tend to be repressible [4], [5], [14]. Similar tendencies have also been found for other physiological functions [15]. We categorized regulated genes into four groups: Catabolism, Anabolism, Transport, and Others, according to their GO and MultiFun [16] classes (Figure 4 and Table S3). We observed that activators correlated with the *holo* conformation and repressors with the *apo* conformation for catabolism (inducible systems), whereas repressors correlated with the *holo* conformation for anabolism (repressible systems), as expected (*P = *4.337×10^−90^). In addition, activators correlated with the *holo* conformation for the Transport class and repressors with the *apo* conformation for the class of Other regulated genes (Figure 1d).

## Discussion

The first contribution of this paper is the gathering of all functional and non functional TF conformations, improving the knowledge of the *E. coli* transcriptional regulatory network. The implications of the results reported here can be interpreted in terms of the demand theory of gene regulation [4], [5]. High demand means that expression levels of target genes are at the high end of their physiological range most of the time in the organism’s natural environment, whereas low demand means that for most of the time expression is at the low end [15], [17]. This theory predicts activator control for a gene whose expression is in high demand and repressor control for a gene whose expression is in low demand. The basis of demand theory is that interference with regulation owing to mutation, errors in transcription or translation, physical influences such as temperature shifts [17], and to noise and nonspecific interactions [18], results in a fitness penalty for activator control in a low-demand environment and for repressor control in a high-demand environment. Although the experimental evidence tends to support these predictions, there are sets of parameter values that could potentially allow for the opposite predictions [4], [5], [19], [20].

Three pieces of knowledge taken together support an interpretation of the observed tendencies in *E. coli* and also suggest to plausible predictions of *apo* and *holo* conformations in other bacteria. The first is the prediction of high or low demand with activation or repression, respectively. The second is the observed correlation of catabolic systems with induction in response to their substrate and of anabolic systems with repression in response to their end product. The third is the assumption, given the data, that TFs in these systems mostly respond to the binding of an effector related to the substrate and end product signals (see the section on “Distribution of TF Functional Conformations in Complex Regulation,” above).

An inducible catabolic system is predicted to use an activator when working under high demand, and if it operates in response to the appearance of the substrate, then the activator has to be in the *holo* conformation (e.g., the maltose operon). A catabolic system in low demand would use a repressor that needs to be functional in the *apo* conformation to induce the system (i.e., the lactose operon). These examples illustrate that catabolic systems in high or low demand would in principle be subject to *holo* activation and *apo* repression, respectively, as shown in Figure 3.

Anabolic machinery, illustrated by amino acid biosynthesis, should work as a repressible system. An environment rich in specific amino acids will rarely require their endogenous synthesis and therefore is a low demand system. *E. coli* often lives in an environment that is relatively rich in several amino acids [15], which correlates with an enriched pool of *holo* repressors. A positive mode of regulation is expected for operons whose expression is frequently required, when the end product of the biosynthetic pathway is seldom present in significant amounts in the organism’s natural environment. *Apo* activators, which might be expected in this context, are rarely found in *E. coli*; the only *apo* activator, Cbl, and the set of 34 *apo*-activating interactions represent the regulation of biosynthetic genes, expected to be in high demand for *E. coli*. It is noteworthy that many of the amino acid and nucleotide biosynthetic pathways in high demand utilize an alternative form of positive regulation, namely, antitermination and attenuation. Examples include the histidine, isoleucine, valine, leucine, methionine, phenylalanine, and threonine systems [15]. It has been predicted that in all amino acids biosynthetic pathways at least one gene is subject to attenuation [21]. The reasons for the use of attenuation for these biosynthetic systems and classical activators for catabolic systems are not well understood. However, even if we were to treat the known attenuator mechanisms as if they represented *apo* activation, there would be only 51 interactions, and so the tendency toward *holo* activation would remain (Figures S12 and S13 and Tables S4 and S5). We can see that the lack of *apo* activation remains a clear tendency, although the rationale for this characterization of the TRN is still unclear.

A corollary of demand theory is the prediction of dual regulation of differentiated cell-specific functions [22]. The dual regulation of the arabinose catabolism system was known to involve a TF, whereas the dual regulation of the tryptophan biosynthesis system was known to involve two different types of mechanisms: a negative mechanism involving a classical repressor and a positive mechanism involving attenuation. The current study shows that dual TFs are very common in *E. coli*. This suggests that a major fraction of cellular functions is selected to meet alternative high and low demands in different environments.

Finally, if one accepts the correlation between mode of transcription control and environmental demand for gene expression [15], then knowledge of the environmental demand can be used to predict the molecular mode of control. Alternatively, knowing the molecular mode of control can be used to predict the correlated feature of the environmental niche of the organism [15]. We have much more complete information available today about the molecular mode of transcription control, as captured in the current version of RegulonDB [1], and this allows us to make numerous predictions about the ecological niche of *E. coli*. The testing of these predictions, however, presents a serious challenge, given the current technology for characterization of heterogeneous microenvironments in complex habitats, such as the mammalian gut or soil.

The major discoveries reported here include the clear dominance of regulators in the *holo* functional conformation and the near absence of activators in the *apo* functional conformation. This observation is true both at the level of TFs, with only 1 (Cbl) of 20 TFs in the activator *apo* conformation, as well as at the level of TF-promoter pairs, with only 34 (3%) undergoing activation in the *apo* conformation among the total of 982 pairs. Given that our analysis covered almost 100 of an estimated total of 300 TFs in *E. coli*, it is tempting to suggest that the observed uneven distribution will remain valid for the complete network, both at the level of TFs and at the level of interactions. Remember for instance the suprising fact that seven promoter sequences were enough for Pribnow to identify the TATA motif of *E. coli* promoters [23], a striking example of conservation of a pattern initially identified in a small sample of a genomic population of elements. However, it might as well be that the currently known TFs may not be an unbiased sampling of all TFs in the genome, particularly as the laboratory conditions differ from the ecological niches of *E. coli.*

Additionally, the TRN shows a tendency for promoters to have at least one interaction in *holo* conformation, most likely a direct consequence of the almost exclusive *holo* functional conformation of global regulators. Finally, we observed a strong dominance of dual (positive and negative) regulation, suggesting that many systems are designed to work under regimens of either high or low demand, depending on different environmental conditions.

These genomic observations, taken together, contribute an important link in the complete sequence of steps that start with an input signal, lead to regulated transcriptional activity, and end in a response that address the initial signal change. Only by completing the characterization of these *gensor units*, as we baptised them [24], will we be able to fully map mechanisms of regulation with physiology and profiles of gene expression changes. The mapping of the physiological mechanisms can be detailed and enriched with information from the large collections of microarrays and other multi-omic levels of knowledge that are now available under a variety of growing conditions [25]. This contribution, together with genomics and the use of demand theory, provides a first step for future studies combining mechanisms and physiology and expression profiles, with the ecology and evolution of *E. coli* in an integrated deciphering of this model organism.

## Methods

### Classification of TFs by their Functional Conformation

We performed an exhaustive analysis of the transcriptional regulatory network of *E. coli* K-12. The different properties analyzed are summarized in Table S1. This table was built using RegulonDB [1] as our primary reference but also with curation of the functional conformations from the published literature (Table S2). The data set of 149 TFs was carefully filtered to include for consideration only experimental evidence, i.e., computational predictions for TFs, promoters, and TF DNA-binding sites (TFBSs) – all individual sites where TFs are bound in DNA – were not included in the analysis. Sigma factors were not considered as TFs in this study.

TFs were classified by their functional conformation into two categories: (i) TFs without an effector or without information about their functional conformation, and (ii) TFs whose effectors and functional conformations have been characterized. The second category was further defined based on the TF’s conformation and the mechanism of regulation, as follows:

a) *holo* activators, e.g., MalT; b) *apo* activators, e.g., Cbl; c) *holo-apo* activators, when the activator can regulate in the *holo* and *apo* conformations, binding to different sites or to the same sites, e.g., ArgP; d) *holo* repressors, e.g., TrpR; e) *apo* repressors, e.g., LacI; f) *holo-apo* repressors, when the repressor can regulate in the *holo* and *apo* conformations, binding to different sites or to the same sites; g) *holo*-dual, TFs that can be activators or repressors but that always regulate in the *holo* conformation, e.g., ArcA; h) *apo*-dual, TFs that can be activators or repressors but that always regulate in the *apo* conformation, e.g., AsnC; i) *holo-apo*-dual, TFs that can be activators or repressors but also can regulate in the *holo* or *apo* conformation, e.g., the arabinose activator protein AraC.

### Classification of TFs Based on the DNA-binding Domain

TFs exhibit a DNA-binding domain that is conserved among evolutionary families. The superfamily and family assignations were based on SUPERFAMILY [26] annotations. We only analyzed families with more than five TFs and evaluated their homogeneity in terms of the functional conformation of their members (*apo*, *holo*, or without effector).

### Classification of the TF-promoter and TF-TFBS Interactions by TF Functional Conformation

TFs interact with different numbers of promoters, e.g., CRP has 210 interactions and LacI has only 3 interactions. Also, the proportion between positive and negative interactions varies, e.g., CRP activates 153 promoters, represses 44, and has 9 dual interactions. Therefore, the TF functional conformation and its effect on the interactions were analyzed. TF-promoter interactions were classified as follows:

a) *holo* activator, e.g., *fucPIKUR*, which is activated by CRP in *holo* conformation; b) *apo* activator, e.g., *tauABCD*, which is activated by Cbl in *apo* conformation; c) *holo* repressor, e.g., *tauABCD*, which is repressed by CysB in *holo* conformation; d) *apo* repressor, e.g., *lacZYA*, which is repressed by LacI in *apo* conformation; e) *holo* dual, e.g., *nagE*, which is both activated and repressed by CRP but only in *holo* conformation; and f) *apo* dual, e.g., *gcvTHP*, which is both activated and repressed by GcvA both in *apo* conformation [1].

In contrast to the classification of TFs, we also can classify dual interactions as activation and repression by the same TF. Some operons can have different promoters, for instance, *lacZYA* has three different promoters. The first one is activated by CRP and so was counted as a *holo* activator; the second promoter is also activated by CRP and was counted as a *holo* activator; the third promoter was counted twice, once for *apo* repression by LacI and once as a *holo* activator for CRP. Note that we counted only one TF-promoter-repressing interaction, even if it involved three operator sites. Thus, we counted four interactions, three of which were counted in the class *holo* activator and one in the *apo* repression class.

Alternatively, we defined the TF-TFBS interactions by counting each binding site as an individual interaction. We performed a similar classification as described for the TF-promoter interactions. At the level of TFBSs, TFs were considered dual only when they activated and repressed the same promoter by using the same DNA-binding site.

For instance, rpoH has four promoters (based on strong experimental evidence). One of these promoters, rpoHp5, has three different TFBSs (two for activation and dual regulation by CRP in holo conformation and one for repression by CytR in apo conformation), and so the TF-promoter interactions at this promoter include some in the holo conformation by CRP and repression in the apo conformation by CytR. For this situation, we counted two interactions with two different conformations. When we analyzed at the level of TF-TFBS interactions, we counted two for CRP, one for activation, and one for dual regulation, both in the holo conformation, and one for CytR repression in the apo conformation.

### Classification of TFs as Global or Local

Based on previous definitions a global regulator is a TF that regulates a large number of genes that participate in several metabolic pathways, and that shows a relatively low clustering coefficient (meaning that its regulated genes rarely regulate themselves) [27]–[29]. Based on these criteria we considered the following to be global TFs: CRP, FNR, IHF, Fis, ArcA, NarL, H-NS, Lrp, FlhDC, and Fur.

### Classification of Functional Conformations of Multiple TFs Regulating Promoters

We classified promoters based on their TF interactions. For instance, the *lacZYA* promoter is activated by CRP (*holo* activator) and repressed by LacI (*apo* repressor). This promoter is classified as a *holo* activator/*apo* repressor combination. We classified all promoters and determined the frequencies of all types of combinations. With this classification, we did not duplicate any promoter, and we included the interactions of TFs without effectors, e.g., those that are regulated by H-NS, which does not need an effector for regulation (see Figure S6).

### Functional Classes of the Regulated Genes

We classified regulated genes according to their functional class, based on MultiFun [16] and ontologies [30]. By classifying the functional conformation of the multiple TFs regulating a promoter, we obtained the *bnumber* of the genes for each promoter. Then, a correlation was made with the functional class in MultiFun. Also, with the corresponding gene ontologies, we categorized the genes into the categories Catabolism, Anabolism, Transport, and Others.

### Regulation by Attenuation

In theory, the relative abundance levels of amino acids in the colon are considered to be in the following order: lysine>glutamate>arginine>tyrosine>tryptophan>glycine>leucine >phenylalanine>histidine>alanine>serine>valine>aspartate >proline>threonine>cysteine>isoleucine>methionine [15]. Based on this ranking, we assume that the demand in the colon for each amino acid can be high or low. We assigned the predicted abundances as described elsewhere [31], since it is known that among this list, those amino acids from lysine to tryptophan are estimated to be abundant [32] and consequently in low demand; the amino acids from leucine to methionine are predicted to be in high demand [15].

It has been predicted that amino acids that are seldom frequent in the colon would be regulated by a positive mode of control [15]. However, there are few activators in repressible systems. Histidine is predicted to be regulated by a positive mode of control; however, it does not have any known TF acting in its biosynthetic pathway. In fact, it is regulated only by attenuation. Thus, it is positive when it is in high demand because the antiterminator is formed and negative when in low demand because of the formation of the terminator. Isoleucine, valine, leucine, methionine, phenylalanine, and threonine appear to be in high demand, and an attenuation system has been described for these amino acids. For alanine, serine, aspartate, proline, and cysteine, we did not find experimental evidence associating these amino acids with attenuation systems. There was an activator that regulated alanine, aspartate, and cysteine, as expected; however, serine was associated with repression and proline was associated with dual control. Briefly, there are seven cases of attenuation described: five cases for amino acids predicted to be in high demand and two cases for amino acids predicted to be in low demand in the colon (Table S3).

If we had to add the attenuation systems to the *apo* activation counts, they contribute much less to the asymmetry in the *apo* vs *holo* activators count, but still a significant under-representation of *apo* activation remains unexplained (Figure S8).

If we take into account the predicted attenuators generated by computational procedures in the genome of *E. coli* K-12 [21], we observed that almost all the amino acids have a system of attenuation, except for glycine (Table S4). Nonetheless, these numbers do not increase the *apo* activation interactions, and so *apo* activation is still underrepresented as shown in Figure S9.

### Statistical Analyses

Chi-square tests for independence were implemented to compare the categorical groups in each analysis. The null hypothesis was that the variables were independent. The alternative hypothesis was that the categorical groups were related. The Yates correction was used when the observed frequency was small. We used an α of 0.05 in our tests. All the tests and graphs were determined using the R program [33].

## Supporting Information

Figure S1 **Asymmetries in the functional conformations of TFs.** Similar to Fig. 1 but with an additional column of those TFs with no effector known. TFs were classified based on the mode of control (activator: green; repressor: red; dual: blue) and the functional conformation (holo, apo, holo-apo, or without effector [no eff]). Pearson’s chi-squared test for the functional conformation and the function of the TF: χ^2^ = 32.2174, df = 6, P = 1.482×10^−05^.
(TIF)

Figure S2 **Heterogeneity of functional conformations within TF families.** TFs were classified based on the SUPERFAMILY classification and the functional conformation (apo, holo, holo-apo, or without effector). Gray portions of bars indicate the fractions of TFs without an effector or with no known effector. All families except TetR contain holo and apo members. The two-component systems covalently modified TFs are mainly in holo conformation. Since the aim of this analysis is to analyze the heterogeneity of conformations within each family, we arbitrarily limited the analysis to families with 5 or more members. Smaller families continue to show heterogeneity in the conformation (data not shown).
(TIF)

Figure S3 **Asymmetries in TF-promoter interactions.** TF-promoter interactions were classified according to the mode of control (activation: green; repression: red; dual: blue) and the functional conformation (holo, apo, holo-apo, or without effector [no eff]) of the TF. Pearson’s chi-squared test: χ^2^ = 88.6169, df = 4, P<2.2×10^−16^.
(TIF)

Figure S4 **Asymmetries in TF-TFBS interactions.** TF-TFBS regulatory interactions (RIs) were classified according to the mode of control (activation: green; repression: red; dual: blue) and the functional conformation (holo, apo, holo-apo, and without effector [no eff]) of the TF. Pearson’s chi-squared test: χ^2^ = 142.479, df = 4, P<2.2×10^−16^.
(TIF)

Figure S5 **Effects of global TFs on promoter interactions with local TFs.** Promoter interactions with global and local TFs were classified. Here we present the local TF-promoter interactions that fall within the scope of a global regulator. They were classified according to the mode of control (activation: green; repression: red; dual: blue) and functional conformation (holo, apo, holo-apo, or without effector [no eff]) of the TF. Pearson’s chi-squared test: χ^2^ = 79.4576, df = 4, P = 2.269×10^−16^.
(TIF)

Figure S6 **Promoter regulation without two-component system TFs.** This figure is to be compared with S3, but in this case we excluded all TF members of the two-component family. Again, promoters were classified according to the mode of control (activation: green; repression: red; dual: blue) and functional conformation (holo, apo, holo-apo, or without effector [no eff]) of the TF. Compare the Pearson’s chi-squared test: χ^2^ = 8.3486, df = 4, P = 0.07961 with that obtained when the two-component systems were included.
(TIF)

Figure S7 **Combinatorial regulation of promoters.** Each bar corresponds to the number of promoters regulated with the given combination. Combinations are defined according to the TF’s mode of control (activation, repression, dual) and the functional conformation (holo, apo, holo-apo, or without effector [–]).Within each such class, we separated by colors the contributions of promoters subject to only one, two, three and four or more TFs.
(TIF)

Figure S8 **Regulation of promoters by one functional conformation.** Each bar corresponds to the number of promoters regulated with the given combination.
(TIF)

Figure S9 **Regulation of promoters by two functional conformations.** Each bar corresponds to the number of promoters regulated with the given combination of two different functional conformations of TFs.
(TIF)

Figure S10 **Regulation of promoters by three functional conformations.** Each bar corresponds to the number of promoters regulated with the given combination of three different functional conformations of TFs. More than three combinations are not shown but most of them have only one case by combination.
(TIF)

Figure S11 **Distribution of promoters regulated by at least one TF in holo conformation.** Each bar corresponds to the relative frequency of promoters regulated by at least one TF in each conformation (holo, apo, or without effector) or at least one TF in more than one conformation (holo and apo, holo and without effector, or apo and without effector).
(TIF)

Figure S12 **Regulation by TFs and by attenuation.** Blue bars correspond to TFs that were classified based on the mode of control (activation, repression or dual) and the functional conformation (holo, apo, holo-apo). Red section correspond to the number of cases in amino acids biosynthesis that have at least one attenuation system. Pearson’s chi-squared test: χ^2^ = 20.1826, df = 4, P = 0.0004596.
(TIF)

Figure S13 **Frequency of interactions by TFs and attenuation.** Blue bars correspond to TF-promoter interactions that were classified according to the mode of control (activation, repression or dual) and the functional conformation (holo, apo, holo-apo) of the TF. Red bars correspond to all the attenuation systems known and predicted for amino acids biosynthesis. Pearson’s chi-squared test: χ^2^ = 76.3451, df = 2, P<2.2×10^−16^.
(TIF)

Table S1 **Properties analyzed.** This table contains the collection of the following properties analyzed: functional conformation, TF mode of control, TF-promoter interactions, TF-TFBSs interaction, TF function in a global regulatory network, promoter regulation, TF evolutionary family, functional class of the regulated genes and attenuation.
(DOCX)

Table S2 **TF dataset.** This table shows the list of TFs; however a recent version of the dataset can be found at http://regulondb.ccg.unam.mx/
(DOCX)

Table S3 **Gene classification based on MultiFun.** This table shows the mapping of MultiFun categories with Catabolism, Anabolism, Transport and Other.
(DOCX)

Table S4 **Transcriptional regulation in amino acid pathways.** Regulation of amino acid pathways by TFs and by attenuation.
(DOCX)

Table S5 **Prediction of attenuators in genes that belong to amino acid biosynthesis pathways.** Predictions obtained from Merino & Yanofsky (2005).
(DOC)

## References

[1] Salgado H, Peralta-Gil M, Gama-Castro S, Santos-Zavaleta A, Muñiz-Rascado L, et al (2013) RegulonDB v8.0: omics data sets, evolutionary conservation, regulatory phrases, cross-validated gold standards and more. Nucleic Acids Research 41: D203–D213. 23203884 10.1093/nar/gks1201 PMC3531196

[2] Gutiérrez-Ríos RM, Rosenblueth DA, Loza JA, Huerta AM, Glasner JD, et al (2003) Regulatory Network of Escherichia coli: Consistency Between Literature Knowledge and Microarray Profiles. Genome Res 13: 2435–2443. 14597655 10.1101/gr.1387003 PMC403762

[3] Wall ME, Hlavacek WS, Savageau MA (2004) Design of gene circuits: lessons from bacteria. Nat Rev Genet 5: 34–42. 14708014 10.1038/nrg1244

[4] Savageau MA (1998) Demand Theory of Gene Regulation. I. Quantitative Development of the Theory. Genetics 149: 1665–1676. 9691027 10.1093/genetics/149.4.1665 PMC1460276

[5] Savageau MA (1998) Demand Theory of Gene Regulation. II. Quantitative Application to the Lactose and Maltose Operons of Escherichia coli. Genetics 149: 1677–1691. 9691028 10.1093/genetics/149.4.1677 PMC1460280

[6] Stec E, Witkowska-Zimny M, Hryniewicz MM, Neumann P, Wilkinson AJ, et al (2006) Structural basis of the sulphate starvation response in E. coli: crystal structure and mutational analysis of the cofactor-binding domain of the Cbl transcriptional regulator. J Mol Biol 364: 309–322. 17010379 10.1016/j.jmb.2006.06.033

[7] Newman EB, Lin R (1995) Leucine-responsive regulatory protein: a global regulator of gene expression in E. coli. Annu Rev Microbiol 49: 747–775. 8561478 10.1146/annurev.mi.49.100195.003531

[8] Ansaldi M, Simon G, Lepelletier M, Mejean V (2000) The TorR high-affinity binding site plays a key role in both torR autoregulation and torCAD operon expression in Escherichia coli. J Bacteriol 182: 961–966. 10648521 10.1128/jb.182.4.961-966.2000 PMC94371

[9] Giedroc DP, Arunkumar AI (2007) Metal sensor proteins: nature’s metalloregulated allosteric switches. Dalton Trans: 3107–3120. 10.1039/b706769k 17637984

[10] Kuriyan J, Eisenberg D (2007) The origin of protein interactions and allostery in colocalization. Nature 450: 983–990. 18075577 10.1038/nature06524

[11] Swint-Kruse L, Matthews KS (2009) Allostery in the LacI/GalR family: variations on a theme. Curr Opin Microbiol 12: 129–137. 19269243 10.1016/j.mib.2009.01.009 PMC2688824

[12] Chen S, Iannolo M, Calvo JM (2005) Cooperative Binding of the Leucine-Responsive Regulatory Protein (Lrp) to DNA. Journal of Molecular Biology 345: 251–264. 15571719 10.1016/j.jmb.2004.10.047

[13] Collado-Vides J, Magasanik B, Gralla JD (1991) Control site location and transcriptional regulation in Escherichia coli. Microbiol Rev 55: 371–394. 1943993 10.1128/mr.55.3.371-394.1991 PMC372825

[14] Savageau MA (1977) Design of molecular control mechanisms and the demand for gene expression. Proc Natl Acad Sci U S A 74: 5647–5651. 271992 10.1073/pnas.74.12.5647 PMC431845

[15] Savageau MA (1989) Are there rules governing patterns of gene regulation? In: Goodwin BC, and Saunders, P.T., editor. Theoretical biology: epigenetic and evolutionary order from complex systems Edinburgh University Press. 42–66.

[16] Serres MH, Riley M (2000) MultiFun, a multifunctional classification scheme for Escherichia coli K-12 gene products. Microb Comp Genomics 5: 205–222. 11471834 10.1089/omi.1.2000.5.205

[17] Savageau MA (1976) Biochemical systems analysis : a study of function and design in molecular biology. Reading, Mass.: Addison-Wesley Pub. Co., Advanced Book Program. xvii, 379 p. p.

[18] Alon U (2007) An introduction to systems biology : design principles of biological circuits. Boca Raton, FL: Chapman & Hall/CRC. xvi, 301 p., [304] p. of plates p.

[19] Gerland U, Hwa T (2009) Evolutionary selection between alternative modes of gene regulation. Proc Natl Acad Sci U S A 106: 8841–8846. 19470486 10.1073/pnas.0808500106 PMC2690017

[20] Ray JCJ, Tabor JJ, Igoshin OA (2011) Non-transcriptional regulatory processes shape transcriptional network dynamics. Nat Rev Micro 9: 817–828. 10.1038/nrmicro2667 PMC3755963 21986901

[21] Merino E, Yanofsky C (2005) Transcription attenuation: a highly conserved regulatory strategy used by bacteria. Trends in Genetics 21: 260–264. 15851059 10.1016/j.tig.2005.03.002

[22] Savageau MA (1983) Regulation of differentiated cell-specific functions. Proc Natl Acad Sci U S A 80: 1411–1415. 6219393 10.1073/pnas.80.5.1411 PMC393607

[23] Pribnow D (1975) Nucleotide sequence of an RNA polymerase binding site at an early T7 promoter. Proc Natl Acad Sci U S A 72: 784–788. 1093168 10.1073/pnas.72.3.784 PMC432404

[24] Gama-Castro S, Salgado H, Peralta-Gil M, Santos-Zavaleta A, Muniz-Rascado L, et al (2011) RegulonDB version 7.0: transcriptional regulation of Escherichia coli K-12 integrated within genetic sensory response units (Gensor Units). Nucleic Acids Res 39: D98–105. 21051347 10.1093/nar/gkq1110 PMC3013702

[25] Engelen K, Fu Q, Meysman P, Sanchez-Rodriguez A, De Smet R, et al (2011) COLOMBOS: access port for cross-platform bacterial expression compendia. PLoS One 6: e20938. 21779320 10.1371/journal.pone.0020938 PMC3136457

[26] de Lima Morais DA, Fang H, Rackham OJ, Wilson D, Pethica R, et al (2011) SUPERFAMILY 1.75 including a domain-centric gene ontology method. Nucleic Acids Res 39: D427–434. 21062816 10.1093/nar/gkq1130 PMC3013712

[27] Freyre-Gonzalez JA, Alonso-Pavon JA, Trevino-Quintanilla LG, Collado-Vides J (2008) Functional architecture of Escherichia coli: new insights provided by a natural decomposition approach. Genome Biol 9: R154. 18954463 10.1186/gb-2008-9-10-r154 PMC2760881

[28] Gottesman S (1984) Bacterial regulation: global regulatory networks. Annu Rev Genet 18: 415–441. 6099091 10.1146/annurev.ge.18.120184.002215

[29] Martínez-Antonio A, Collado-Vides J (2003) Identifying global regulators in transcriptional regulatory networks in bacteria. Curr Opin Microbiol 6: 482–489. 14572541 10.1016/j.mib.2003.09.002

[30] Ashburner M, Ball CA, Blake JA, Botstein D, Butler H, et al (2000) Gene ontology: tool for the unification of biology. The Gene Ontology Consortium. Nat Genet 25: 25–29. 10802651 10.1038/75556 PMC3037419

[31] Savageau MA (1979) Autogenous and classical regulation of gene expression: a general theory and experimental evidence. In: Goldberger AL, editor. Biological regulation and development. New York: Plenum Press. 57–100.

[32] Savageau MA (1983) Escherichia coli habitats, cell types, and molecular mechanisms of gene control. The american naturalist 122: 732–744.

[33] R Development Core Team (2011) R: A language and environment for statistical computing. R Foundation for Statistical Computing, Vienna, Austria. ISBN 3–900051–07–0, URL.

