# Correction: Transcription Factors in *Escherichia coli* Prefer the *Holo* Conformation

**DOI:** 10.1371/annotation/96d7b9a4-aa2e-4593-ae8a-7c14d134e29c

**Published:** 2013-08-22

**Authors:** Yalbi Itzel Balderas-Martínez, Michael Savageau, Heladia Salgado, Ernesto Pérez-Rueda, Enrique Morett, Julio Collado-Vides

There was an error in the Funding statement. The correct version of the statement is available below:

YIB-M acknowledges the PDCB of CCG-UNAM and her support by a PhD fellowship (228320/210360) from CONACyT-México. This research was partially supported by CCG-UNAM, by grant CB-103686 from CONACYT to JC-V, and grants 5R01GM071962-08 to JC-V and RO1GM030054-23 to MAS from the National Institute of General Medical Sciences, National Institutes of Health, USA. The content is solely the responsibility of the authors and does not necessarily represent the official views of the National Institute of General Medical Sciences or the National Institutes of Health. EP-R was partially supported by DGAPA grant IN209511. The funders had no role in study design, data collection and analysis, decision to publish, or preparation of the manuscript. 

