# Supplementary figures and images for: Transcription Factors in Escherichia coli Prefer the Holo Conformation
Source: PLoS One. 2013 Jun 12;8(6):e65723. doi: 10.1371/journal.pone.0065723 (PMC3680503; doi:10.1371/journal.pone.0065723)

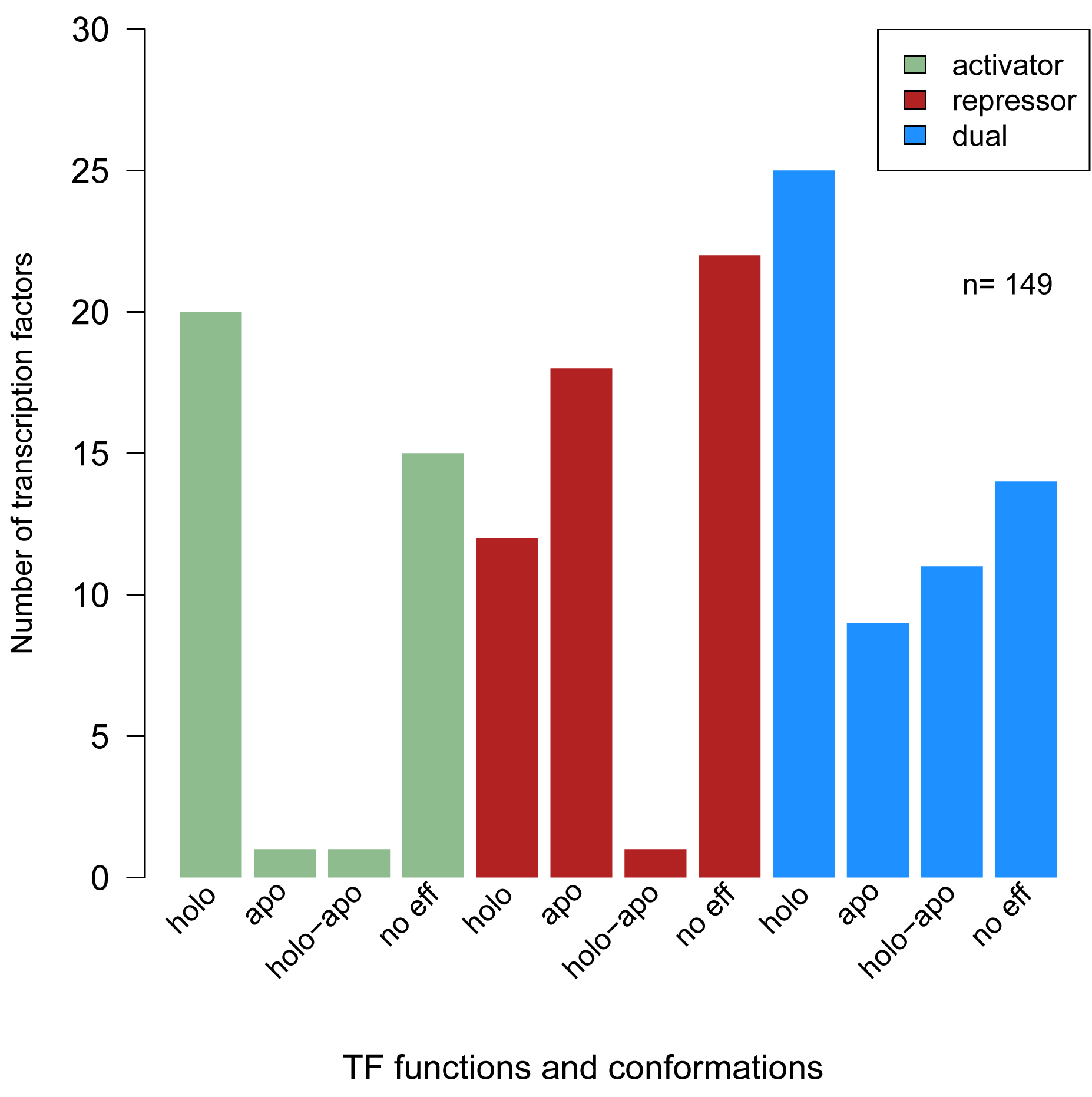

Supplement: Figure S1 — Asymmetries in the functional conformations of TFs. Similar to Fig. 1 but with an additional column of those TFs with no effector known. TFs were classified based on the mode of control (activator: green; repressor: red; dual: blue) and the functional conformation (holo, apo, holo-apo, or without effector [no eff]). Pearson’s chi-squared test for the functional conformation and the function of the TF: χ2 = 32.2174, df = 6, P = 1.482×10^−05^. (TIF) [file pone.0065723.s001.tif]

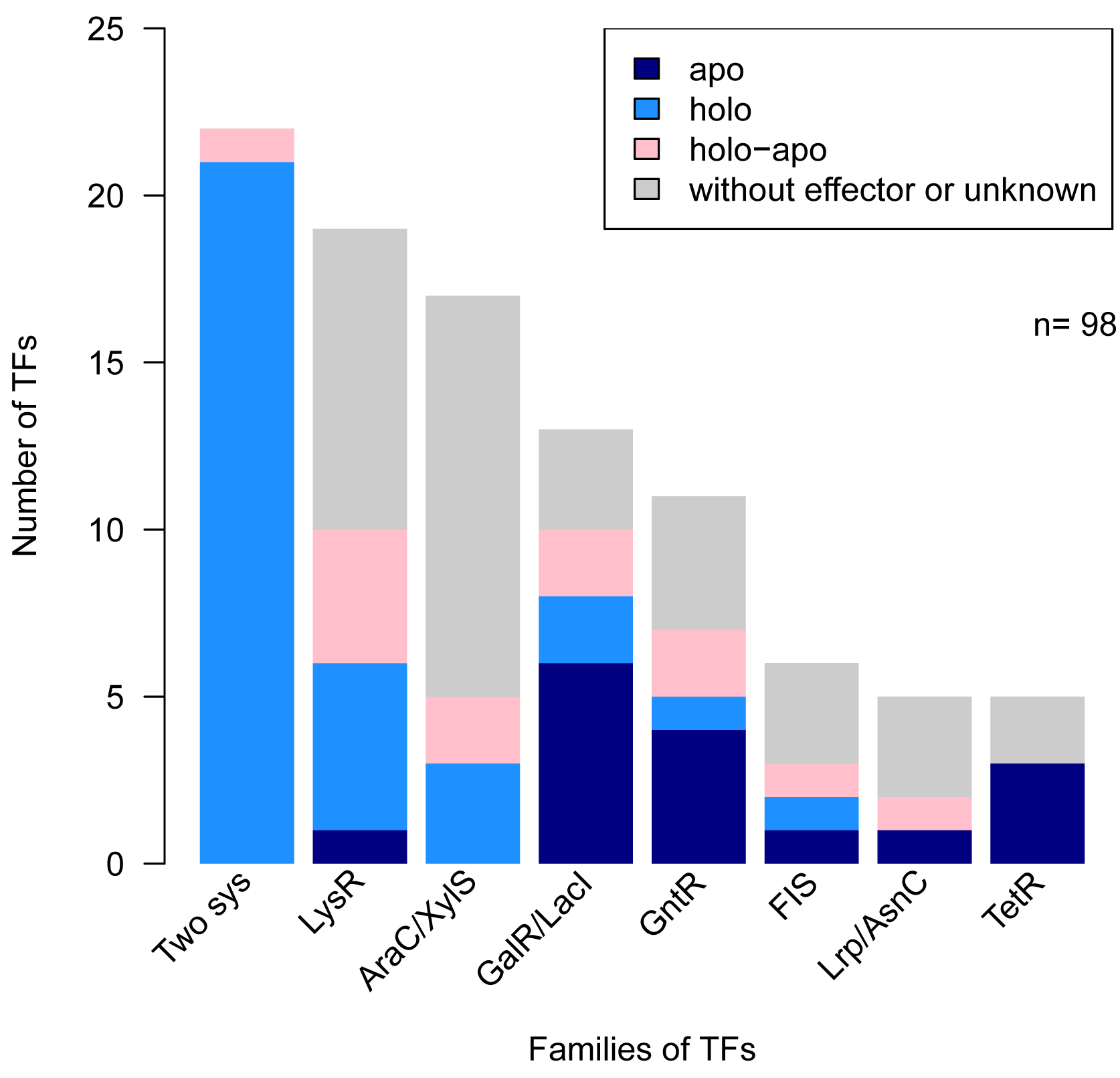

Supplement: Figure S2 — Heterogeneity of functional conformations within TF families. TFs were classified based on the SUPERFAMILY classification and the functional conformation (apo, holo, holo-apo, or without effector). Gray portions of bars indicate the fractions of TFs without an effector or with no known effector. All families except TetR contain holo and apo members. The two-component systems covalently modified TFs are mainly in holo conformation. Since the aim of this analysis is to analyze the heterogeneity of conformations within each family, we arbitrarily limited the analysis to families with 5 or more members. Smaller families continue to show heterogeneity in the conformation (data not shown). (TIF) [file pone.0065723.s002.tif]

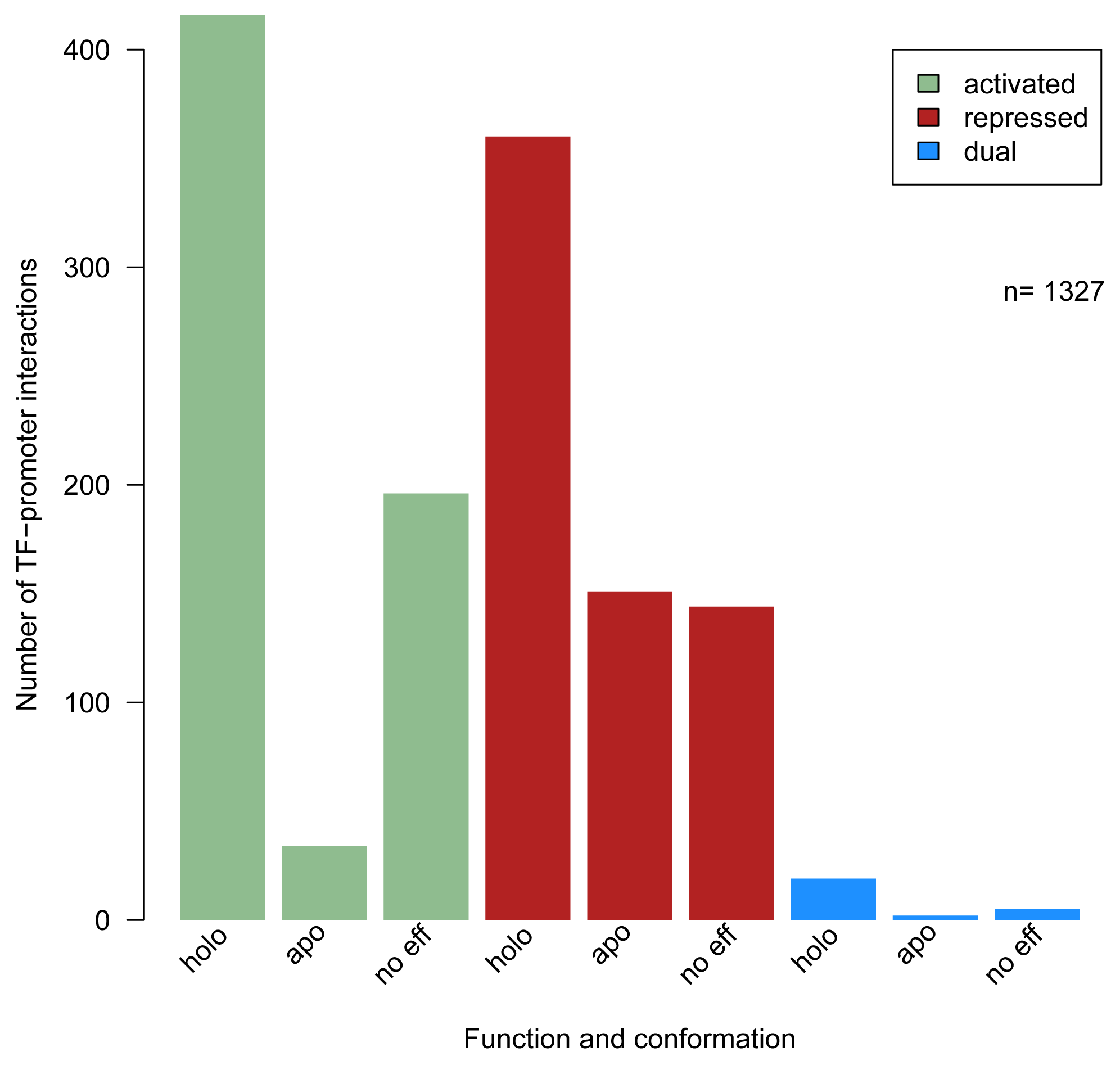

Supplement: Figure S3 — Asymmetries in TF-promoter interactions. TF-promoter interactions were classified according to the mode of control (activation: green; repression: red; dual: blue) and the functional conformation (holo, apo, holo-apo, or without effector [no eff]) of the TF. Pearson’s chi-squared test: χ2 = 88.6169, df = 4, P<2.2×10^−16^. (TIF) [file pone.0065723.s003.tif]

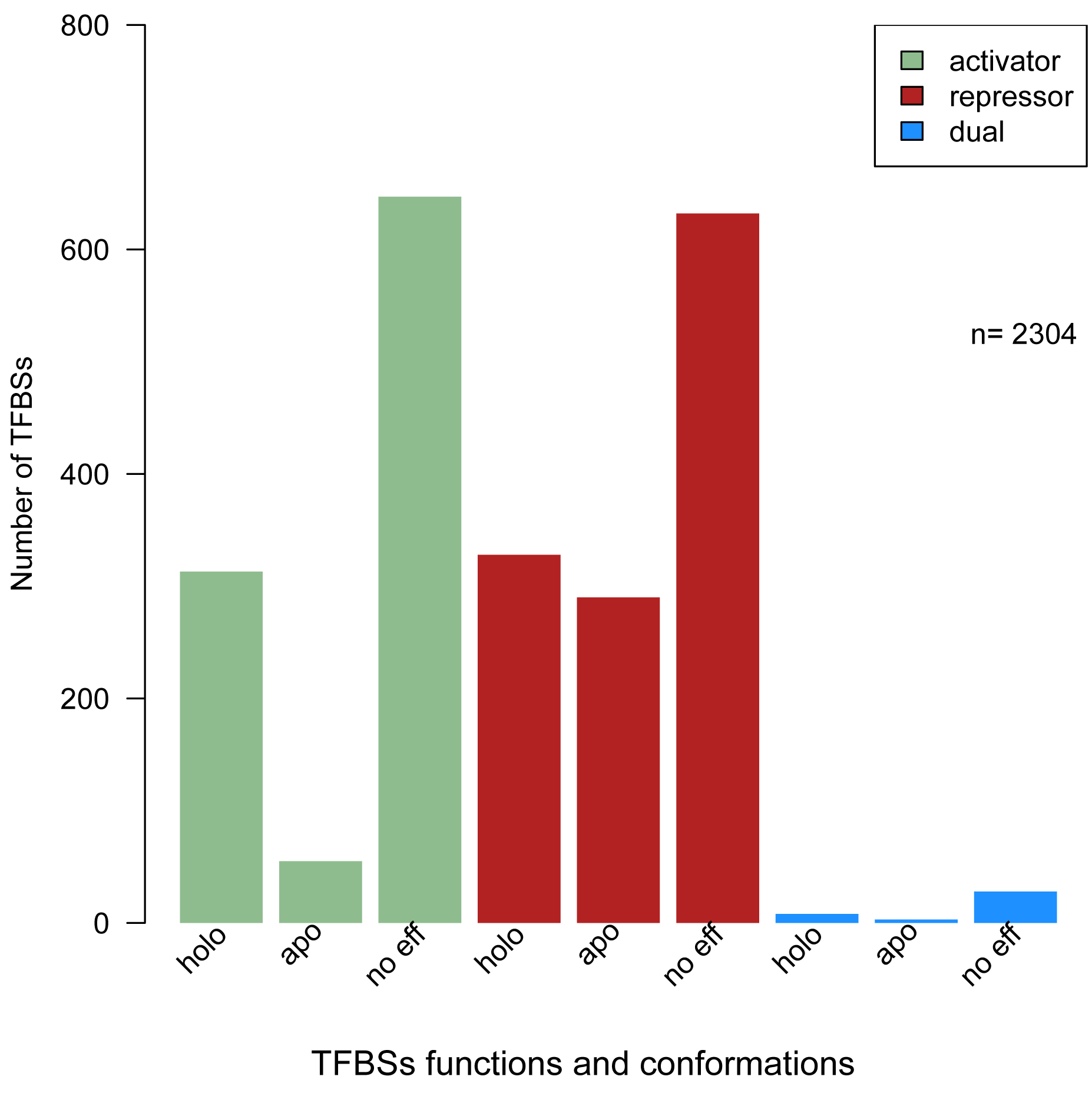

Supplement: Figure S4 — Asymmetries in TF-TFBS interactions. TF-TFBS regulatory interactions (RIs) were classified according to the mode of control (activation: green; repression: red; dual: blue) and the functional conformation (holo, apo, holo-apo, and without effector [no eff]) of the TF. Pearson’s chi-squared test: χ2 = 142.479, df = 4, P<2.2×10^−16^. (TIF) [file pone.0065723.s004.tif]

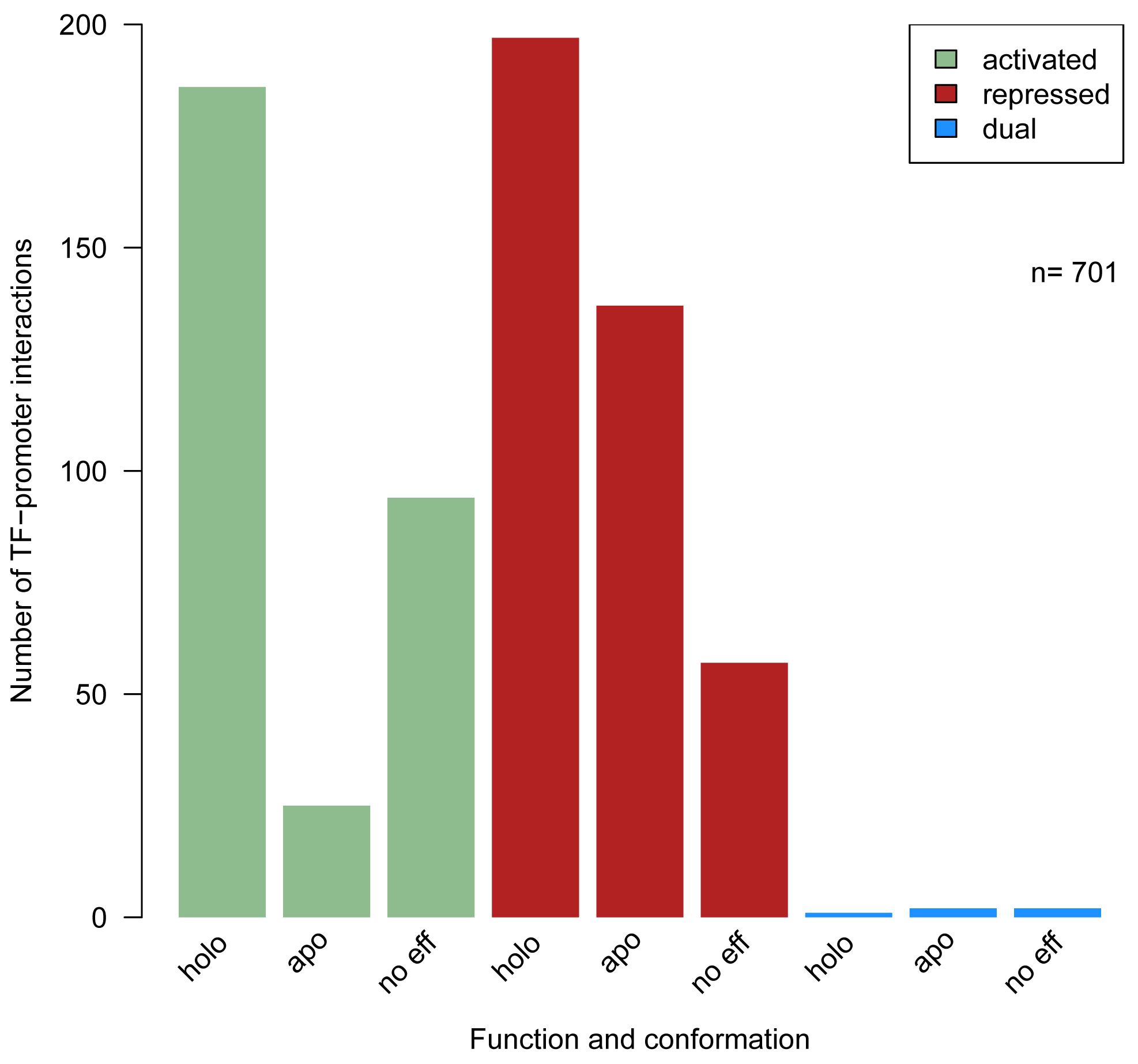

Supplement: Figure S5 — Effects of global TFs on promoter interactions with local TFs. Promoter interactions with global and local TFs were classified. Here we present the local TF-promoter interactions that fall within the scope of a global regulator. They were classified according to the mode of control (activation: green; repression: red; dual: blue) and functional conformation (holo, apo, holo-apo, or without effector [no eff]) of the TF. Pearson’s chi-squared test: χ2 = 79.4576, df = 4, P = 2.269×10^−16^. (TIF) [file pone.0065723.s005.tif]

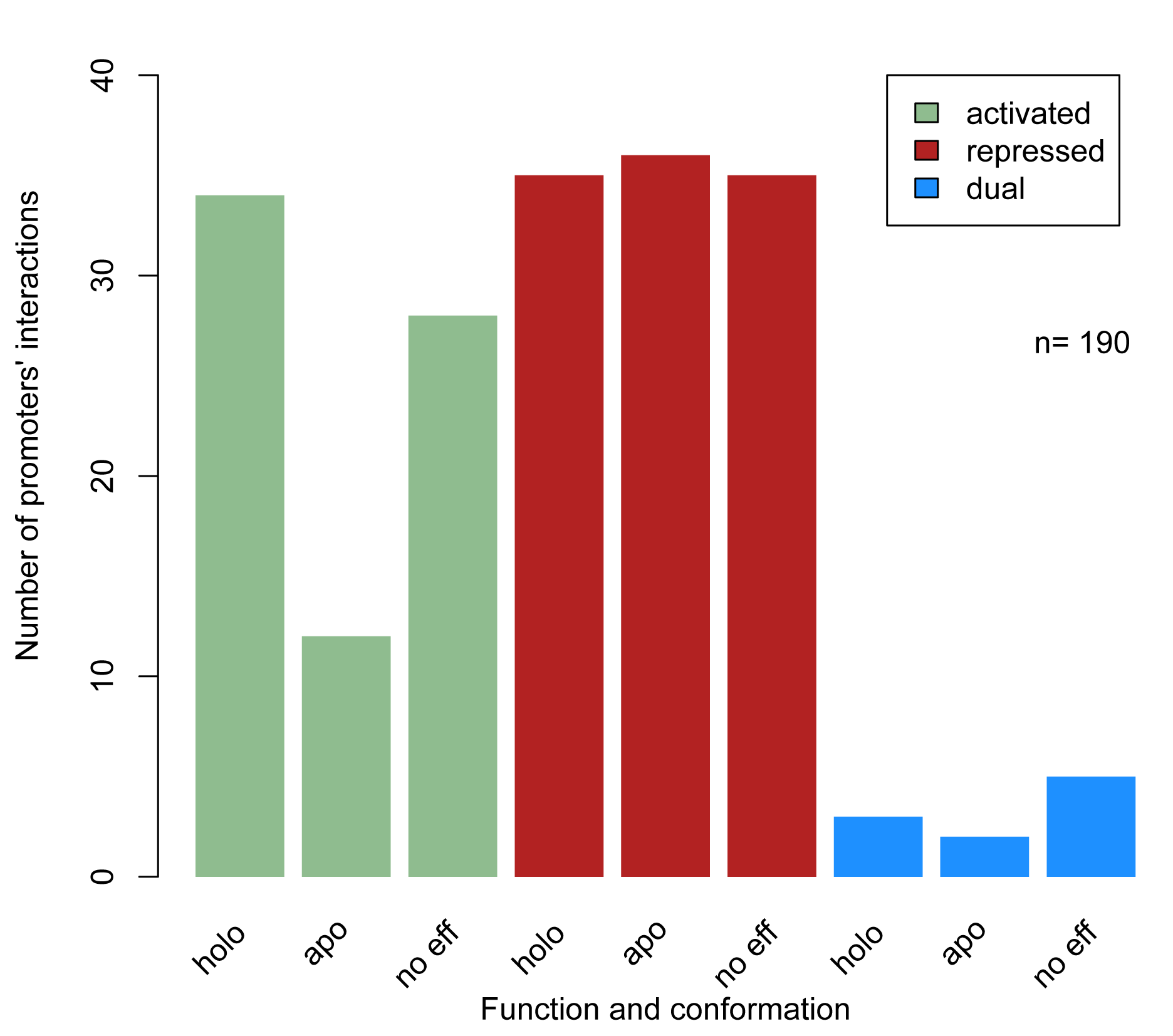

Supplement: Figure S6 — Promoter regulation without two-component system TFs. This figure is to be compared with S3, but in this case we excluded all TF members of the two-component family. Again, promoters were classified according to the mode of control (activation: green; repression: red; dual: blue) and functional conformation (holo, apo, holo-apo, or without effector [no eff]) of the TF. Compare the Pearson’s chi-squared test: χ2 = 8.3486, df = 4, P = 0.07961 with that obtained when the two-component systems were included. (TIF) [file pone.0065723.s006.tif]

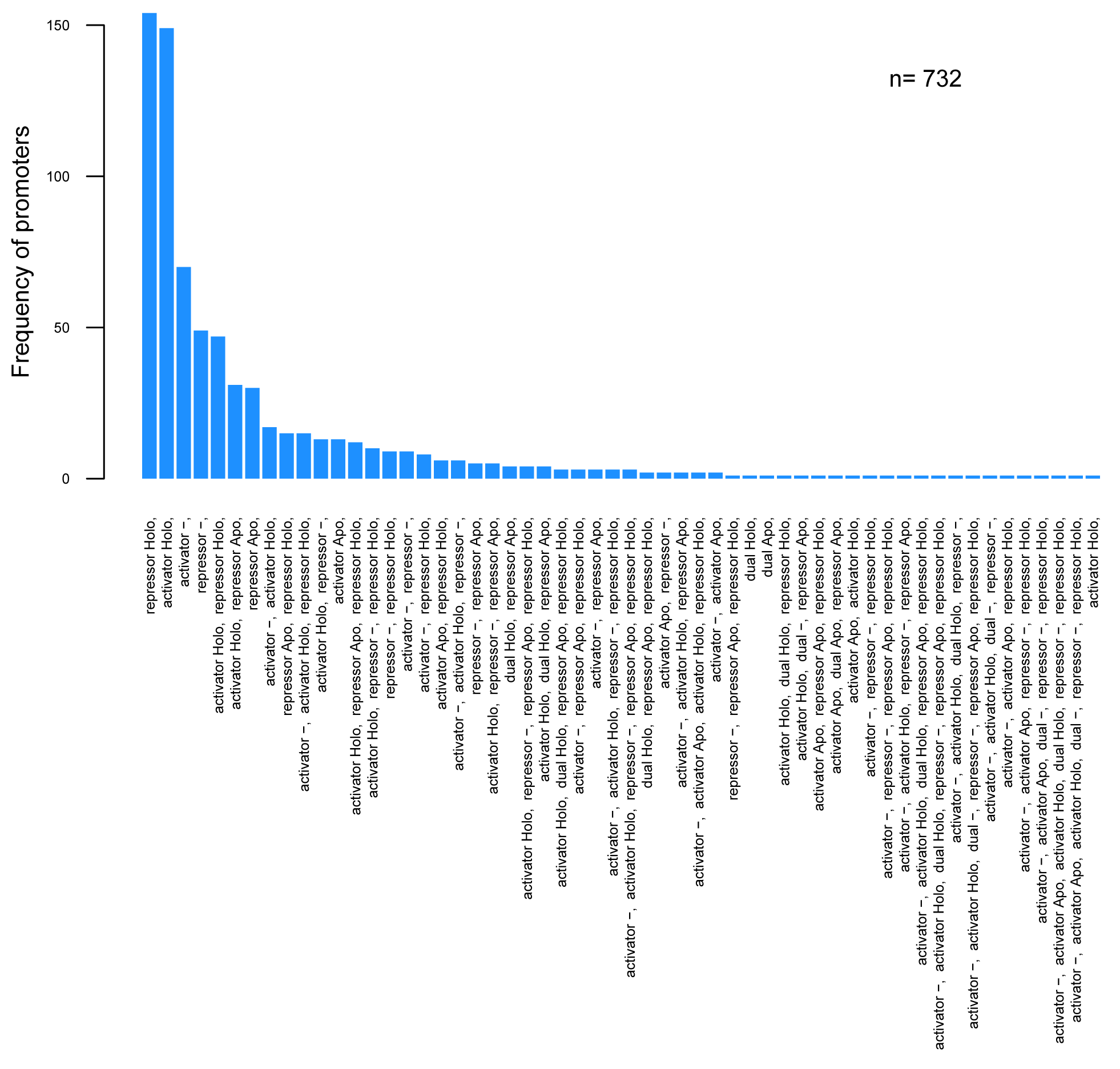

Supplement: Figure S7 — Combinatorial regulation of promoters. Each bar corresponds to the number of promoters regulated with the given combination. Combinations are defined according to the TF’s mode of control (activation, repression, dual) and the functional conformation (holo, apo, holo-apo, or without effector [–]).Within each such class, we separated by colors the contributions of promoters subject to only one, two, three and four or more TFs. (TIF) [file pone.0065723.s007.tif]

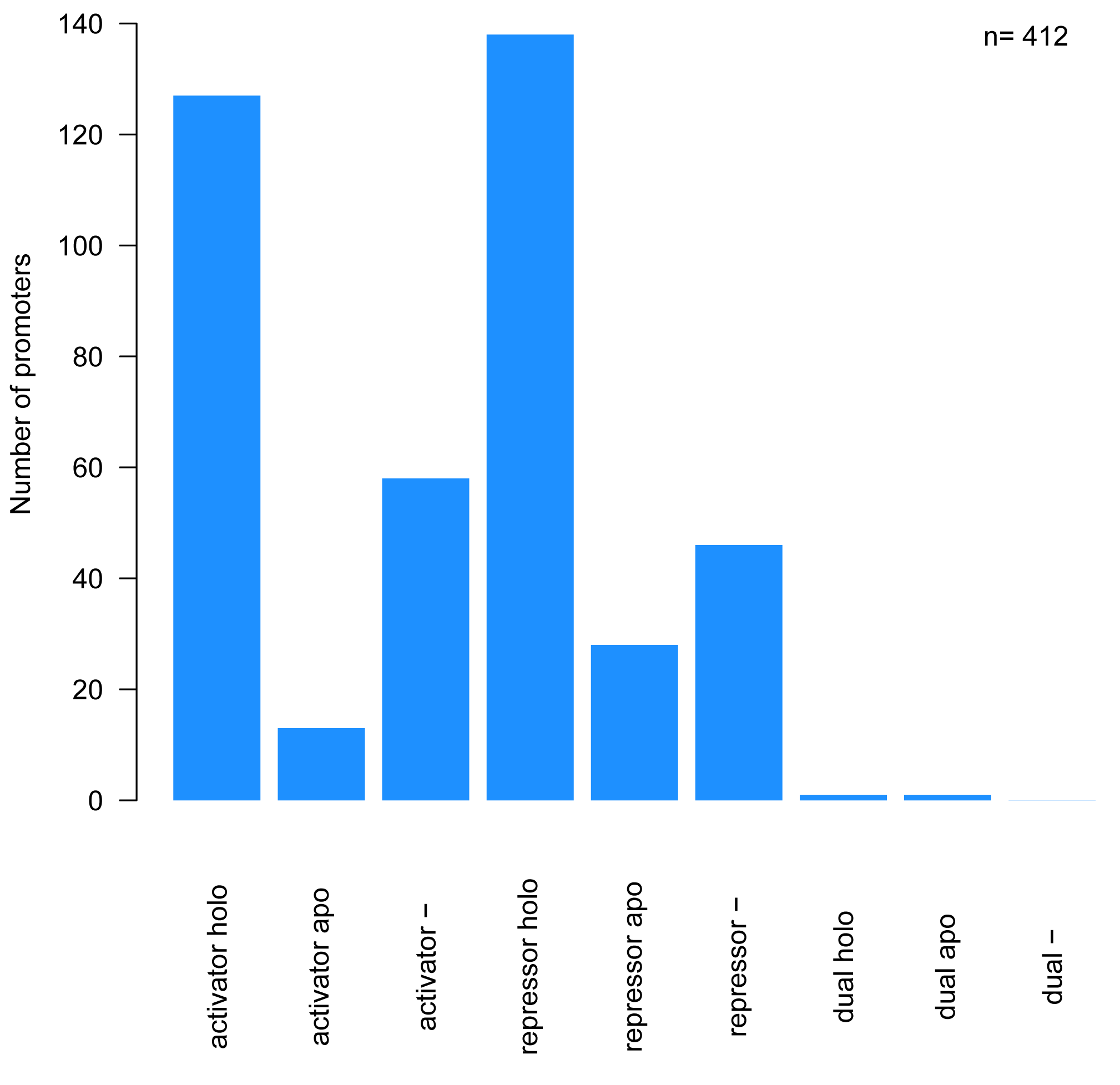

Supplement: Figure S8 — Regulation of promoters by one functional conformation. Each bar corresponds to the number of promoters regulated with the given combination. (TIF) [file pone.0065723.s008.tif]

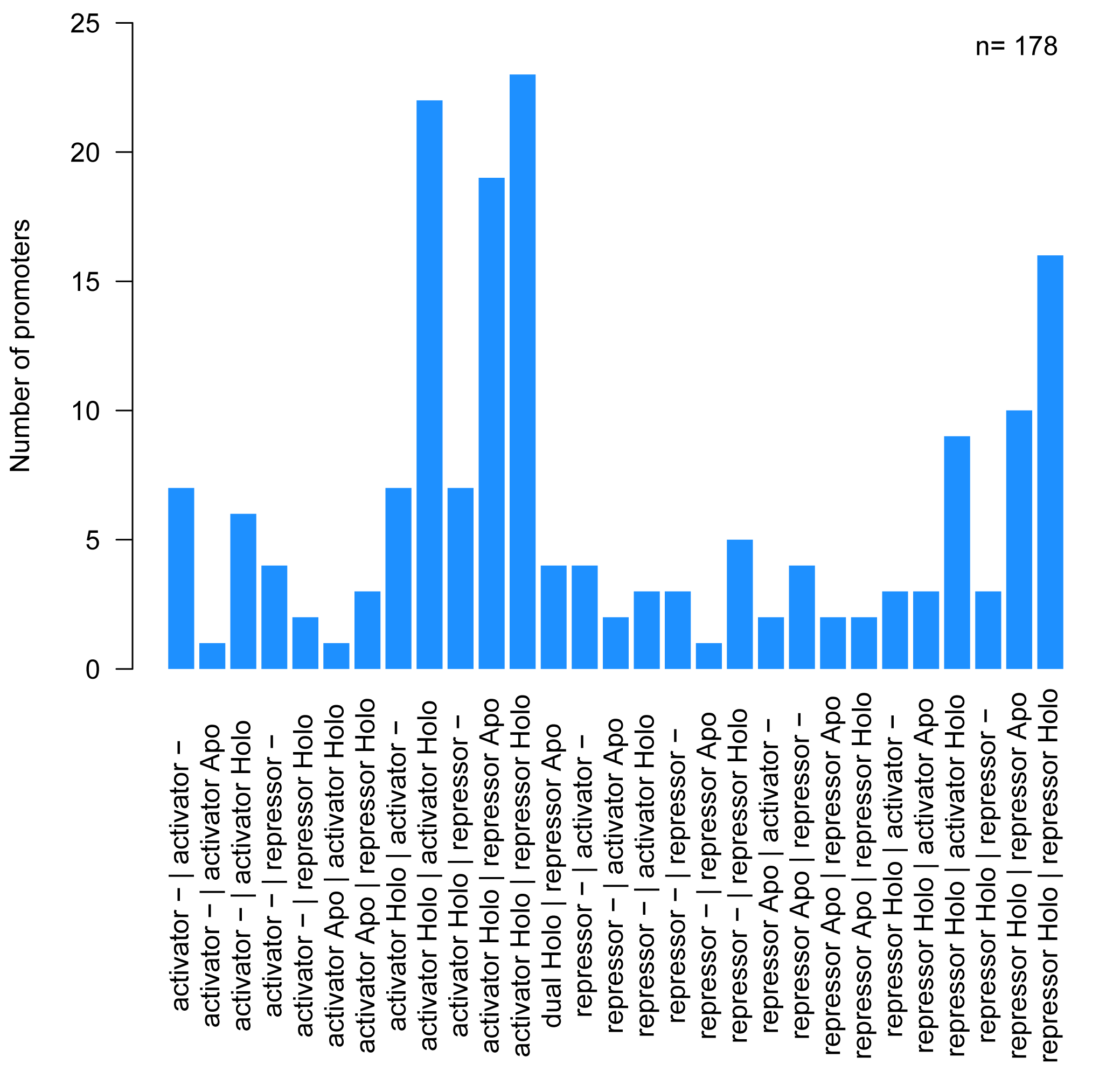

Supplement: Figure S9 — Regulation of promoters by two functional conformations. Each bar corresponds to the number of promoters regulated with the given combination of two different functional conformations of TFs. (TIF) [file pone.0065723.s009.tif]

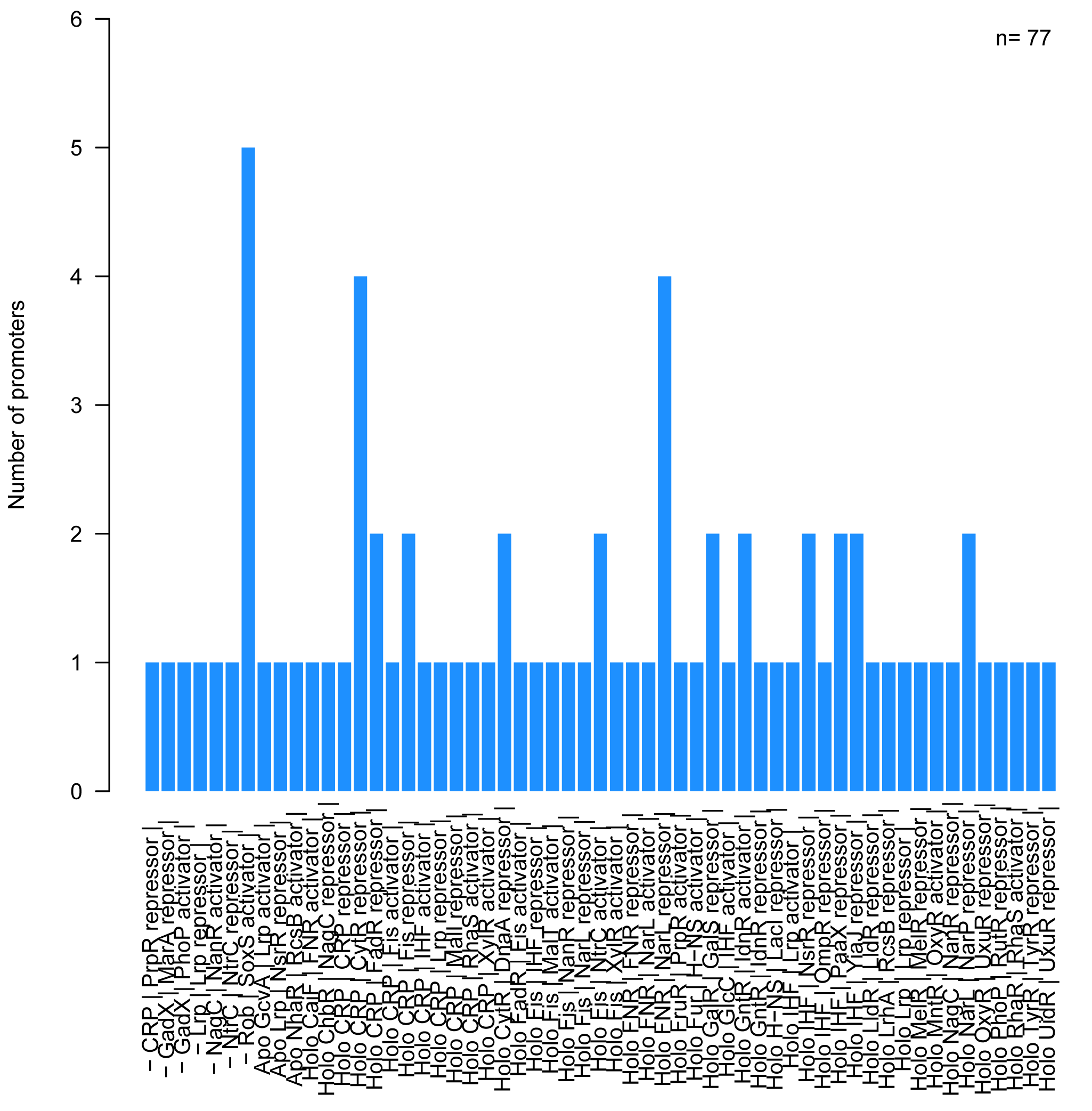

Supplement: Figure S10 — Regulation of promoters by three functional conformations. Each bar corresponds to the number of promoters regulated with the given combination of three different functional conformations of TFs. More than three combinations are not shown but most of them have only one case by combination. (TIF) [file pone.0065723.s010.tif]

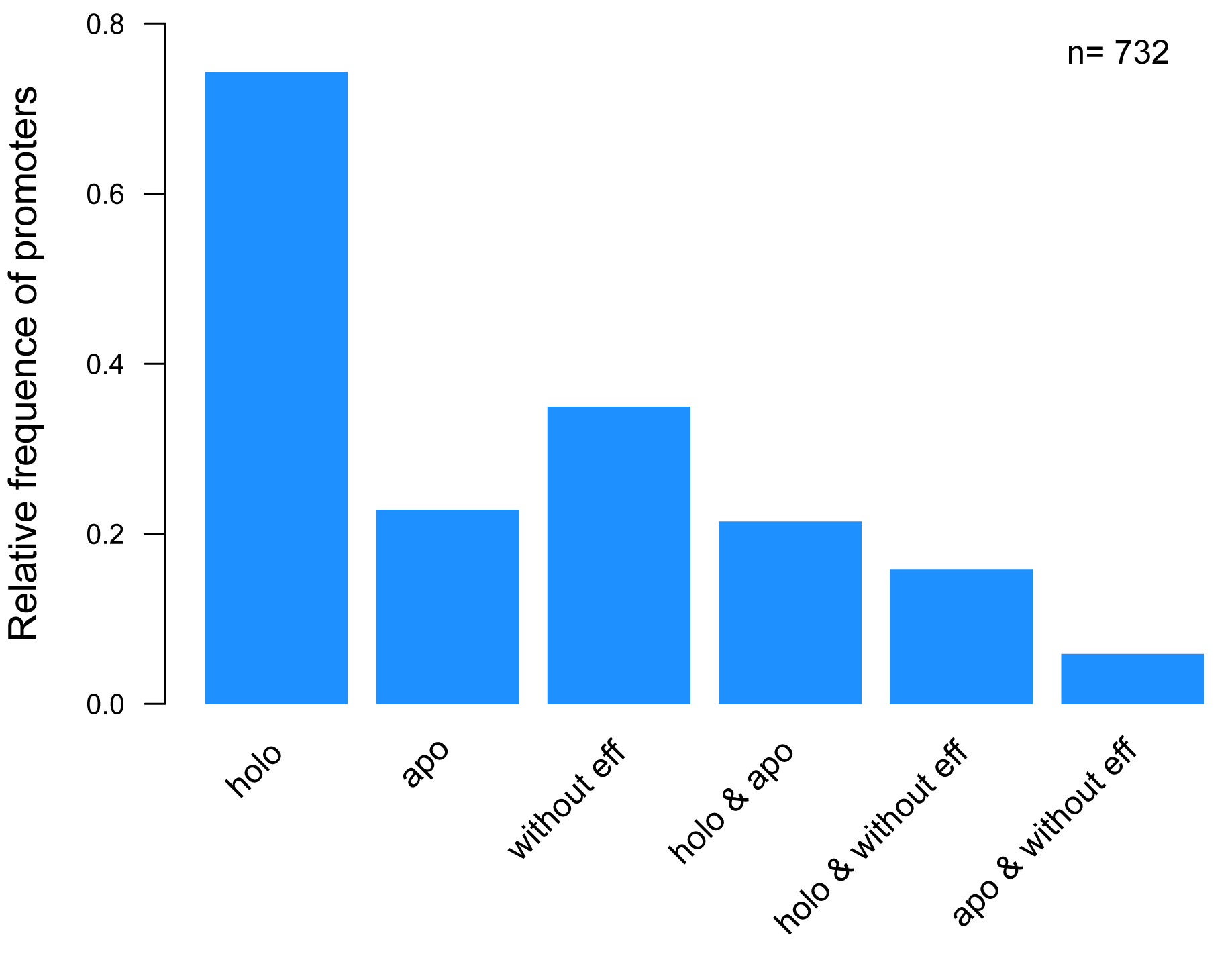

Supplement: Figure S11 — Distribution of promoters regulated by at least one TF in holo conformation. Each bar corresponds to the relative frequency of promoters regulated by at least one TF in each conformation (holo, apo, or without effector) or at least one TF in more than one conformation (holo and apo, holo and without effector, or apo and without effector). (TIF) [file pone.0065723.s011.tif]

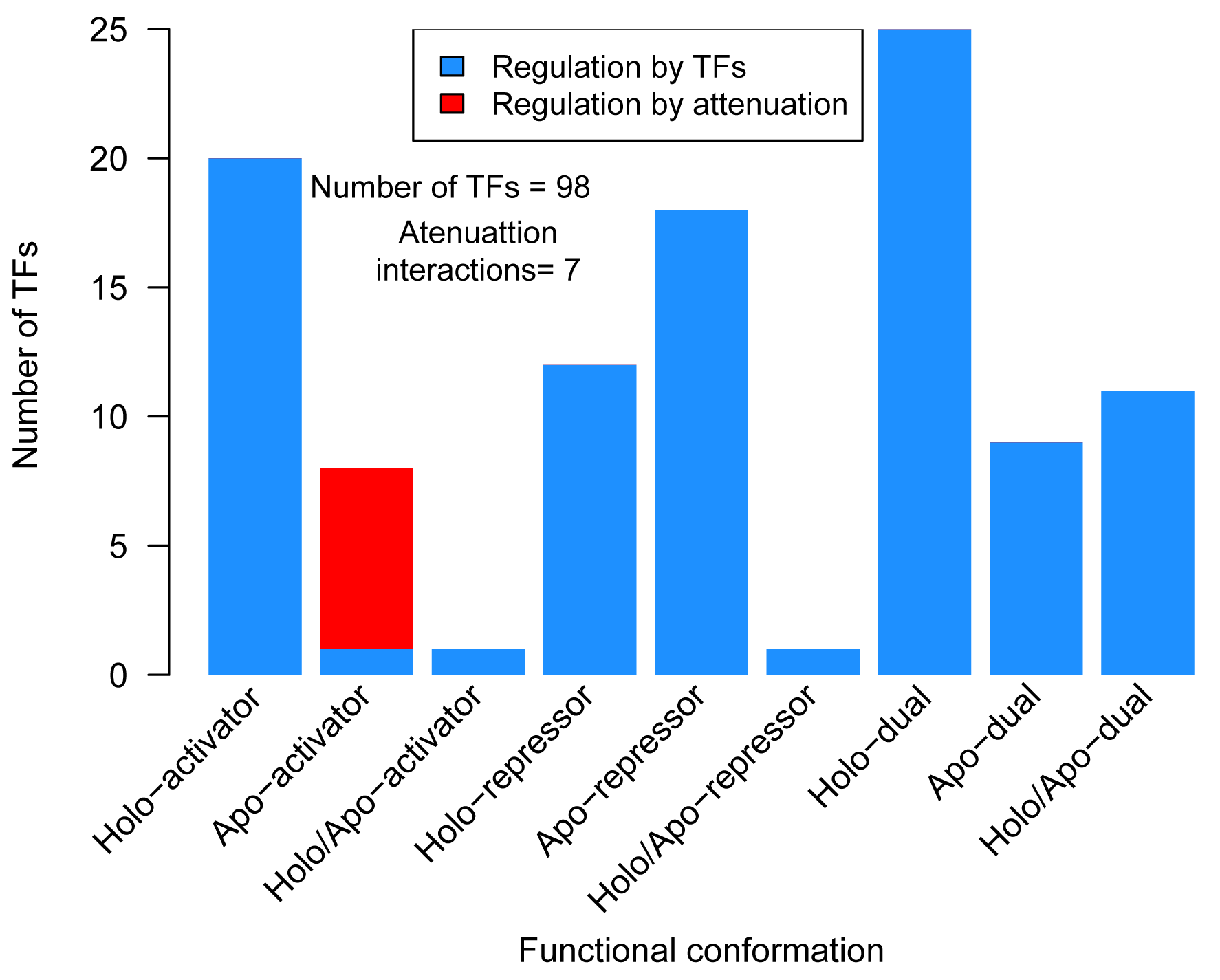

Supplement: Figure S12 — Regulation by TFs and by attenuation. Blue bars correspond to TFs that were classified based on the mode of control (activation, repression or dual) and the functional conformation (holo, apo, holo-apo). Red section correspond to the number of cases in amino acids biosynthesis that have at least one attenuation system. Pearson’s chi-squared test: χ2 = 20.1826, df = 4, P = 0.0004596. (TIF) [file pone.0065723.s012.tif]

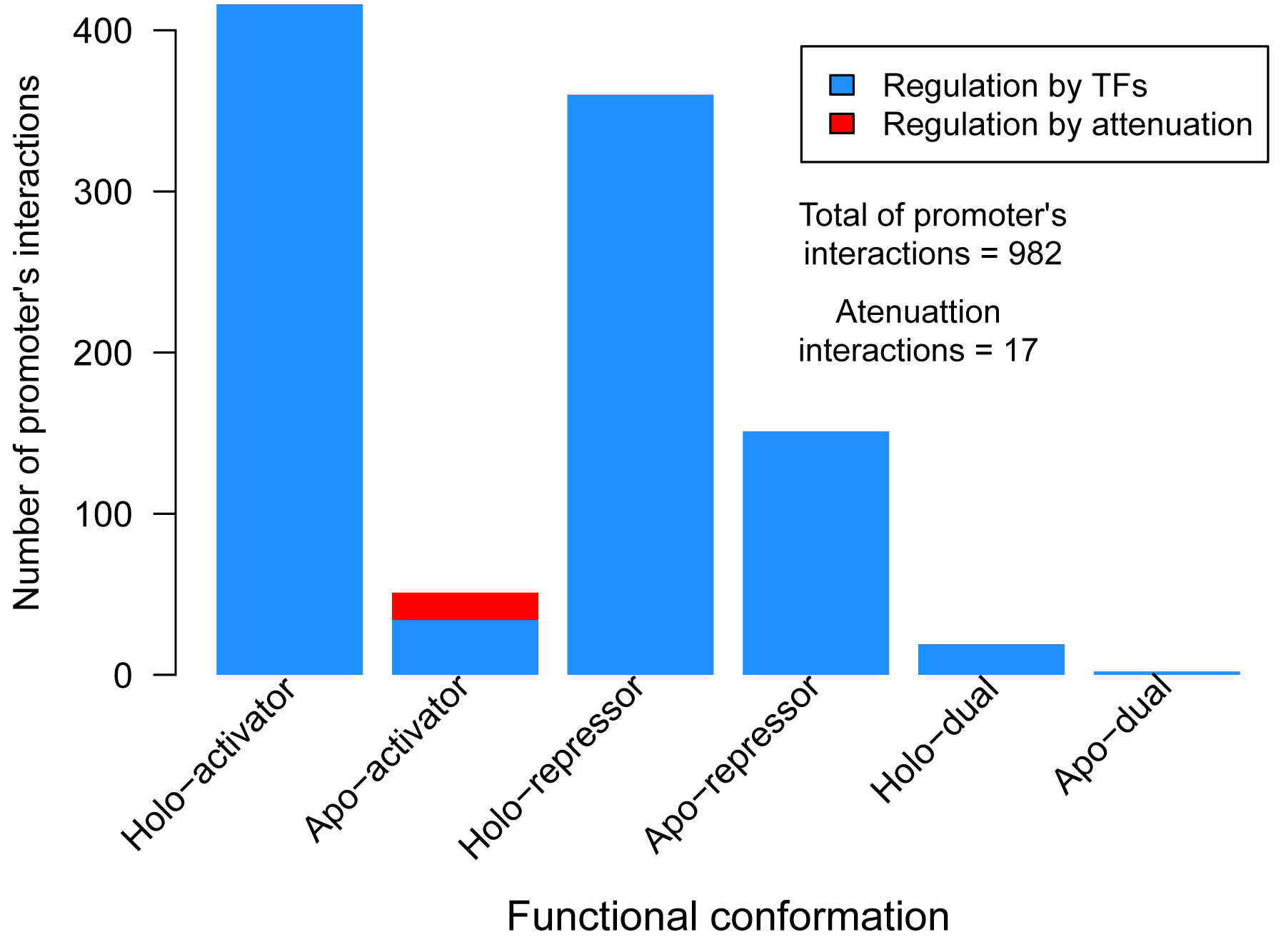

Supplement: Figure S13 — Frequency of interactions by TFs and attenuation. Blue bars correspond to TF-promoter interactions that were classified according to the mode of control (activation, repression or dual) and the functional conformation (holo, apo, holo-apo) of the TF. Red bars correspond to all the attenuation systems known and predicted for amino acids biosynthesis. Pearson’s chi-squared test: χ2 = 76.3451, df = 2, P<2.2×10^−16^. (TIF) [file pone.0065723.s013.tif]
