# Supplementary material for: Transcription Factors in Escherichia coli Prefer the Holo Conformation
Source: PLoS One. 2013 Jun 12;8(6):e65723. doi: 10.1371/journal.pone.0065723 (PMC3680503; doi:10.1371/journal.pone.0065723)
Supplement: Table S1 — Properties analyzed. This table contains the collection of the following properties analyzed: functional conformation, TF mode of control, TF-promoter interactions, TF-TFBSs interaction, TF function in a global regulatory network, promoter regulation, TF evolutionary family, functional class of the regulated genes and attenuation. (DOCX) [file pone.0065723.s014.docx]

**Table S1. Properties analyzed**

| TF property | Classes | Data source |
| --- | --- | --- |
| Functional conformation | *holo*, *apo*, *holo-apo*, or without effector | [[1](#_ENREF_1)], EcoTFs (http://ecotfs.lanl.gov) [[2](#_ENREF_2)], EcoCyc (http://ecocyc.org/)[[3](#_ENREF_3)], this work (http://regulondb.ccg.unam.mx) |
| TF mode of control | Activation, repression, dual | RegulonDB [[4](#_ENREF_4)] (http://regulondb.ccg.unam.mx) |
| TF-promoter interactions | Activation, repression, dual | RegulonDB [[4](#_ENREF_4)] (http://regulondb.ccg.unam.mx) |
| TF-TFBS interactions | Activation, repression, dual | RegulonDB [[4](#_ENREF_4)] (http://regulondb.ccg.unam.mx) |
| TF function in a global regulatory network | Global, local | [[5](#_ENREF_5)], [[6](#_ENREF_6)] |
| Promoter regulation | Different combinations by TF mode (activation, repression, dual) and TF conformation (*apo*, *holo*, or without effector) | This work (http://regulondb.ccg.unam.mx) |
| TF evolutionary family | Superfamily classification | Superfamily [[7](#_ENREF_7)] |
| Functional class of the regulated genes | Evaluation with two different classifications | MultiFun [[8](#_ENREF_8)], GO’s [[9](#_ENREF_9)], this work (http://regulondb.ccg.unam.mx) |
| Attenuation | Analysis of a dataset that contains predictions of attenuators | [[10](#_ENREF_10)] and also EcoCyc [[3](#_ENREF_3)] when there is an experiment associated |

^[[1]](#footnote-1)^

**REFERENCES**

1. Gutiérrez-Ríos RM, Rosenblueth DA, Loza JA, Huerta AM, Glasner JD, et al. (2003) Regulatory Network of Escherichia coli: Consistency Between Literature Knowledge and Microarray Profiles. Genome Res 13: 2435-2443.

2. Wall ME, Hlavacek WS, Savageau MA (2004) Design of gene circuits: lessons from bacteria. Nat Rev Genet 5: 34-42.

3. Keseler IM, Collado-Vides J, Santos-Zavaleta A, Peralta-Gil M, Gama-Castro S, et al. (2011) EcoCyc: a comprehensive database of Escherichia coli biology. Nucleic Acids Res 39: D583-590.

4. Salgado H, Peralta-Gil M, Gama-Castro S, Santos-Zavaleta A, Muñiz-Rascado L, et al. (2013) RegulonDB v8.0: omics data sets, evolutionary conservation, regulatory phrases, cross-validated gold standards and more. Nucleic Acids Research 41: D203-D213.

5. Martínez-Antonio A, Collado-Vides J (2003) Identifying global regulators in transcriptional regulatory networks in bacteria. Curr Opin Microbiol 6: 482-489.

6. Freyre-Gonzalez JA, Alonso-Pavon JA, Trevino-Quintanilla LG, Collado-Vides J (2008) Functional architecture of Escherichia coli: new insights provided by a natural decomposition approach. Genome Biol 9: R154.

7. de Lima Morais DA, Fang H, Rackham OJ, Wilson D, Pethica R, et al. (2011) SUPERFAMILY 1.75 including a domain-centric gene ontology method. Nucleic Acids Res 39: D427-434.

8. Serres MH, Riley M (2000) MultiFun, a multifunctional classification scheme for Escherichia coli K-12 gene products. Microb Comp Genomics 5: 205-222.

9. Ashburner M, Ball CA, Blake JA, Botstein D, Butler H, et al. (2000) Gene ontology: tool for the unification of biology. The Gene Ontology Consortium. Nat Genet 25: 25-29.

10. Merino E, Yanofsky C (2005) Transcription attenuation: a highly conserved regulatory strategy used by bacteria. Trends in Genetics 21: 260-264.

1. TF = Transcription Factor; TFBS = Transcription Factor Binding Site [↑](#footnote-ref-1)
