# Supplementary material for: Transcription Factors in Escherichia coli Prefer the Holo Conformation
Source: PLoS One. 2013 Jun 12;8(6):e65723. doi: 10.1371/journal.pone.0065723 (PMC3680503; doi:10.1371/journal.pone.0065723)
Supplement: Table S2 — TF dataset. This table shows the list of TFs; however a recent version of the dataset can be found at http://regulondb.ccg.unam.mx/ (DOCX) [file pone.0065723.s015.docx]

**Table S2. TF data set**

| RegulonDB ID | TF name | Positive effector*^a^* | Negative effector*^b^* | Functional conformation | Type of binding |
| --- | --- | --- | --- | --- | --- |
| ECK120015994 | AcrR |  | R6G, ethidium, proflavin | *apo* | Allosteric |
| ECK120012595 | Ada | Methyl |  | *holo* | Covalent |
| ECK120012515 | AgaR |  |  |  | Without effector |
| ECK125134683 | AidB |  |  |  |  |
| ECK120030264 | AlaS | l-Alanine |  | *holo* | Allosteric |
| ECK120015630 | AllR |  | Glyoxylate | *apo* | Allosteric |
| ECK120012316 | AraC | l-arabinose, d-fucose |  | *holo*-*apo* | Allosteric |
| ECK120011345 | ArcA | Phosphate |  | *holo* | Covalent |
| ECK120011202 | ArgP | l-Arginine | Lysine, canavanine | *holo*-*apo* | Allosteric |
| ECK120011670 | ArgR | l-Arginine |  | *holo* | Allosteric |
| ECK120030724 | ArsR |  | Arsenite, Sb, Bi | *apo* | Allosteric |
| ECK120026318 | AscG |  |  |  |  |
| ECK120013010 | AsnC |  | L-Asparagine | *apo* | Allosteric |
| ECK120013014 | AtoC | Phosphate |  | *holo* | Covalent |
| ECK120015687 | BaeR | Phosphate |  | *holo* | Covalent |
| ECK120013017 | BetI |  | Choline | *apo* | Allosteric |
| ECK120034503 | BglJ |  |  |  |  |
| ECK120012646 | BirA | Bio-5'-AMP |  | *holo* | Allosteric |
| ECK120013805 | CaiF |  |  |  |  |
| ECK120013750 | Cbl |  | Adenosine 5'-phosphosulphate, Thiosulphate | *apo* | Allosteric |
| ECK120012036 | ChbR |  |  |  |  |
| ECK120011210 | CpxR | Phosphate |  | *holo* | Covalent |
| ECK120035002 | CreB | Phosphate |  | *holo* | Covalent |
| ECK120011235 | CRP | cAMP |  | *holo* | Allosteric |
| ECK120013342 | CsgD |  |  |  |  |
| ECK120011587 | CueR | Cu, Ag, Au |  | *holo* | Allosteric |
| ECK120011941 | CusR | Phosphate |  | *holo* | Covalent |
| ECK120013065 | CynR | Cyanate |  | *holo*-*apo* | Allosteric |
| ECK120011312 | CysB | *O*-Acetyl-l-serine | Thiosulfate, sulphide | *holo* | Allosteric |
| ECK120012407 | CytR |  | Cytidine | *apo* | Allosteric |
| ECK120051451 | Dan |  |  |  |  |
| ECK120011348 | DcuR | Phosphate |  | *holo* | Covalent |
| ECK120012664 | DeoR |  | Deoxyribose-5-phosphate | *apo* | Allosteric |
| ECK120011240 | DgsA |  | EIIGlc | *apo* | Allosteric |
| ECK120011903 | DnaA | ATP |  | *holo* | Covalent |
| ECK120035026 | EnvR |  |  |  |  |
| ECK120011575 | EvgA | Phosphate |  | *holo* | Covalent |
| ECK120012141 | ExuR |  |  |  |  |
| ECK120011354 | FabR |  |  |  |  |
| ECK120011824 | FadR |  | Acyl-CoA | *apo* | Allosteric |
| ECK120011394 | FhlA | Formate |  | *holo* | Allosteric |
| ECK120011186 | Fis |  |  |  |  |
| ECK120011332 | FlhDC |  |  |  |  |
| ECK120011229 | FNR | [4Fe-4S] reduced | [2Fe-2S] oxidized | *holo* | Covalent |
| ECK120013112 | FruR |  | Fructose-1,6-diphosphate, fructose-1-phosphate | *apo* | Allosteric |
| ECK120033465 | Fur | Fe^2+^, Mn^2+^ |  | *holo* | Allosteric |
| ECK120012092 | GadE |  |  |  |  |
| ECK120011465 | GadW |  |  |  |  |
| ECK120011458 | GadX |  |  |  |  |
| ECK120012713 | GalR |  | Galactose | *holo*-*apo* | Allosteric |
| ECK120012704 | GalS |  | Galactose | *holo*-*apo* | Allosteric |
| ECK120012465 | GcvA |  | Glycine, purine | *apo* | Allosteric |
| ECK120011437 | GlcC | Glycolate |  | *holo* | Allosteric |
| ECK120012730 | GlpR |  | Glycerol phosphate | *apo* | Allosteric |
| ECK125108627 | GlrR | Phosphate |  | *holo* | Covalent |
| ECK120012096 | GntR |  | Gluconate | *apo* | Allosteric |
| ECK120011294 | H-NS |  |  |  |  |
| ECK120015243 | HcaR |  |  |  |  |
| ECK120026325 | HipB |  |  |  |  |
| ECK120011874 | HU |  |  |  |  |
| ECK120011390 | HyfR |  |  |  |  |
| ECK120011897 | IclR | Pyruvate | Glyoxylate | *holo* | Allosteric |
| ECK120012101 | IdnR | Idonate |  | *holo* | Allosteric |
| ECK120011224 | IHF |  |  |  |  |
| ECK120012965 | IlvY | α-Acetolactate, α-acetohydroxybutyrate |  | *holo*-*apo* | Allosteric |
| ECK120011429 | IscR | [2Fe-2S] ^1+^ |  | *holo* | Covalent |
| ECK120015250 | KdgR |  |  |  |  |
| ECK120012758 | KdpE | Phosphate |  | *holo* | Covalent |
| ECK120012762 | LacI |  | Allolactose | *apo* | Allosteric |
| ECK120013164 | LeuO |  |  |  |  |
| ECK120012770 | LexA | RecA (indirectly) |  | *apo* | Allosteric |
| ECK120013830 | LldR | l-Lactate |  | *holo-apo* | Allosteric |
| ECK120011328 | LrhA |  |  |  |  |
| ECK120011383 | Lrp | Leucine | Alanine | *holo*-*apo* | Allosteric |
| ECK120015681 | LsrR |  | 4,5-dihydroxy-2,3-pentanedione | *apo* | Allosteric |
| ECK120013406 | MalI |  |  |  |  |
| ECK120012218 | MalT | ATP, maltotriose, ADP | MalK, MalY, Aes | *holo* | Allosteric |
| ECK120011412 | MarA |  |  |  |  |
| ECK120013191 | MarR |  | Salicylate, tetracycline, chloramphenicol, plumbagin, dinitrophenol, menadione, TktA, others with phenolic rings | *apo* | Allosteric |
| ECK120051441 | McbR |  |  |  |  |
| ECK120012347 | MelR | Melibiose |  | *holo*-*apo* | Allosteric |
| ECK120011472 | MetJ | *S*-Adenosylmethionine |  | *holo* | Allosteric |
| ECK120012802 | MetR | Homocysteine |  | *holo*-*apo* | Allosteric |
| ECK120011482 | MhpR | 3-(3-hydroxyphenyl)propionate |  | *holo* | Allosteric |
| ECK125134665 | MlrA |  |  |  |  |
| ECK120011949 | MngR |  |  |  |  |
| ECK120015588 | MntR | Mn^2+^ |  | *holo* | Allosteric |
| ECK120011220 | ModE | MoO_4_^2−^ |  | *holo* | Allosteric |
| ECK120011593 | MprA |  | 2,4-Dinitrophenol, Carbonyl cyanide *m*-chlorophenylhydrazone, carbonyl cyanide *p*-(trifluoro-methoxy)phenylhydrazone, | *apo* | Allosteric |
| ECK120051460 | MqsA |  |  |  |  |
| ECK120048826 | MurR |  | MurNac-6-P | *apo* | Allosteric |
| ECK120011265 | Nac |  |  |  |  |
| ECK120013202 | NadR |  |  |  |  |
| ECK120011908 | NagC |  | GlcNac | *apo* | Allosteric |
| ECK120012559 | NanR |  | *N*-Acetyl-neuraminic acid | *apo* | Allosteric |
| ECK120011502 | NarL | Phosphate |  | *holo* | Covalent |
| ECK120011508 | NarP | Phosphate |  | *holo* | Covalent |
| ECK120035042 | NemR |  | *N*-Ethylmaleimide | *apo* | Allosteric |
| ECK120011378 | NhaR | Na^+^ |  | *holo* | Allosteric |
| ECK120011761 | NikR | Ni |  | *holo* | Allosteric |
| ECK120011938 | NorR |  |  |  |  |
| ECK120033053 | NrdR | ATP/dATP and Zn^2+^ |  | *holo* | Allosteric |
| ECK120016814 | NsrR | [2Fe-2S] reduced | Oxidized | *holo*-*apo* | Covalent |
| ECK120011269 | NtrC | Phosphate |  | *holo* | Covalent |
| ECK120011579 | OmpR | Phosphate |  | *holo* | Covalent |
| ECK120011302 | OxyR | Reduced, oxidized |  | *holo* | Covalent |
| ECK120011563 | PaaX |  |  |  |  |
| ECK120012246 | PdhR |  | Pyruvate | *apo* | Allosteric |
| ECK120020634 | PepA |  |  |  |  |
| ECK120011306 | PhoB | Phosphate |  | *holo* | Covalent |
| ECK120011492 | PhoP | Phosphate |  | *holo* | Covalent |
| ECK120015234 | PrpR | 2-Methylcitrate |  | *holo*-*apo* | Allosteric |
| ECK120011400 | PspF |  |  |  |  |
| ECK120012854 | PurR | Hypoxanthine, guanine |  | *holo* | Allosteric |
| ECK120012029 | PutA |  |  |  |  |
| ECK120012277 | QseB | Phosphate |  | *holo* | Covalent |
| ECK120013229 | RbsR |  | d-Ribose | *apo* | Allosteric |
| ECK120033010 | RcnR |  | Ni, Co | *apo* | Allosteric |
| ECK120011324 | RcsB | Phosphate |  | *holo* | Covalent |
| ECK120048934 | RelEB |  |  |  |  |
| ECK120012877 | RhaR | l-Rhamnose |  | *holo* | Allosteric |
| ECK120012337 | RhaS | l-Rhamnose |  | *holo* | Allosteric |
| ECK120011190 | Rob |  |  |  |  |
| ECK120033066 | RstA | Phosphate |  | *holo* | Covalent |
| ECK120033905 | RutR |  | Uracyl, thymine | *apo* | Allosteric |
| ECK120011856 | SdiA |  |  |  |  |
| ECK120032161 | SgrR |  |  |  |  |
| ECK120012253 | SlyA |  |  |  |  |
| ECK120013242 | SoxR | [2Fe-2S]^3+^ oxidized | [2Fe-2S]2^+^ reduced | *holo* | Covalent |
| ECK120011298 | SoxS |  |  |  |  |
| ECK120013255 | TdcA |  |  |  |  |
| ECK120013259 | TdcR |  |  |  |  |
| ECK120012482 | TorR | Phosphate | TorI | *holo*-*apo* | Covalent |
| ECK120011406 | TreR |  | Trealose-6 phosphate | *apo* | Allosteric |
| ECK120012176 | TrpR | l-Tryptophan, |  | *holo* | Allosteric |
| ECK120011194 | TyrR | l-Tryptophan, Phenylalanine, tyrosine |  | *holo*-*apo* | Allosteric |
| ECK120011741 | UhpA | Phosphate |  | *holo* | Covalent |
| ECK120012153 | UidR |  |  |  |  |
| ECK120012165 | UlaR |  |  |  |  |
| ECK120012145 | UxuR |  | Fructuronate | *apo* | Allosteric |
| ECK120013780 | XapR | Xanthosine |  | *holo* | Allosteric |
| ECK120013786 | XylR | Xylose |  | *holo* | Allosteric |
| ECK120048749 | YefM |  |  |  |  |
| ECK120014044 | YiaJ |  |  |  |  |
| ECK125110189 | YqhC |  |  |  |  |
| ECK125134855 | YqjI | Ni, Fe^2+^ |  | *apo* | Allosteric |
| ECK120012024 | ZntR | Zn^2+^, Cd, Hg |  | *holo* | Allosteric |
| ECK120014103 | ZraR | Phosphate |  | *holo* | Covalent |
| ECK120012020 | Zur | Zn^2+^ |  | *holo* | Allosteric |

*^a^*Positive effector: molecule that binds to the TF to change it to a functional conformation.

*^b^*Negative effector: molecule that binds to the TF to change it to a nonfunctional conformation.
