# Supplementary material for: Transcription Factors in Escherichia coli Prefer the Holo Conformation
Source: PLoS One. 2013 Jun 12;8(6):e65723. doi: 10.1371/journal.pone.0065723 (PMC3680503; doi:10.1371/journal.pone.0065723)
Supplement: Table S3 — Gene classification based on MultiFun. This table shows the mapping of MultiFun categories with Catabolism, Anabolism, Transport and Other. (DOCX) [file pone.0065723.s016.docx]

**Table S3. Gene classification based on MultiFun**

| **MultiFun Id** | **Categories** | **Our classification** |
| --- | --- | --- |
| 1 | Metabolism | Catabolism |
| 1.1 | Carbon compound utilization | Catabolism |
| 1.1.1 | Carbohydrates/Carbon compounds | Catabolism |
| 1.1.1.1 | D-allose catabolism | Catabolism |
| 1.1.1.2 | 2,5-ketogluconate metabolism | Catabolism |
| 1.1.1.3 | D-arabinose catabolism | Catabolism |
| 1.1.1.4 | D-galactarate catabolism | Catabolism |
| 1.1.1.5 | D- galacturonate catabolism | Catabolism |
| 1.1.1.6 | D-glucarate catabolism | Catabolism |
| 1.1.1.7 | D-glucuronate catabolism | Catabolism |
| 1.1.1.8 | L-arabinose catabolism | Catabolism |
| 1.1.1.9 | L-idonate catabolism | Catabolism |
| 1.1.1.10 | L-lyxose metabolism | Catabolism |
| 1.1.1.11 | Fucose catabolism | Catabolism |
| 1.1.1.12 | Galactitol catabolism | Catabolism |
| 1.1.1.13 | D-galactonate catabolism | Catabolism |
| 1.1.1.14 | Lactose degradation | Catabolism |
| 1.1.1.15 | Mannose catabolism | Catabolism |
| 1.1.1.16 | Rhamnose catabolism | Catabolism |
| 1.1.1.17 | Sorbitol degradation | Catabolism |
| 1.1.1.18 | Trehalose degradation, low osmolarity+ | Catabolism |
| 1.1.1.19 | Xylose catabolism | Catabolism |
| 1.1.1.20 | Glycol degradation | Catabolism |
| 1.1.1.21 | Mannitol degradation | Catabolism |
| 1.1.1.22 | Ribose degradation | Catabolism |
| 1.1.1.23 | Galactose degradation | Catabolism |
| 1.1.1.24 | Sorbose degradation | Catabolism |
| 1.1.1.25 | L-ascorbate degradation | Catabolism |
| 1.1.1.26 | 2-O-alpha-mannosyl-D-glycerate degradation | Catabolism |
| 1.1.1.27 | Trehalose degradation, high osmolarity | Catabolism |
| 1.1.1.28 | L-galactonate catabolism | Catabolism |
| 1.1.1.29 | Malate utilization | Catabolism |
| 1.1.2 | Fatty acids (fatty acid oxidation) | Catabolism |
| 1.1.2.2 | 3-phenylpropionate and 3-(3-hydroxyphenyl)propionate degradation | Catabolism |
| 1.1.2.3 | Propionate degradation | Catabolism |
| 1.1.2.4 | Anaerobic fatty acid oxidation pathway | Catabolism |
| 1.1.2.5 | Propionyl-CoA (methylmalonyl pathway) | Catabolism |
| 1.1.2.6 | Aerobic fatty acid oxidation pathway | Catabolism |
| 1.1.2.7 | Acetoacetate degradation | Catabolism |
| 1.1.3 | Amino acids | Catabolism |
| 1.1.3.1 | L-alanine degradation | Catabolism |
| 1.1.3.2 | L-serine degradation | Catabolism |
| 1.1.3.3 | Arginine degradation II | Catabolism |
| 1.1.3.4 | Glutamate degradation | Catabolism |
| 1.1.3.5 | Glycine cleavage | Catabolism |
| 1.1.3.6 | Proline utilization | Catabolism |
| 1.1.3.7 | Threonine catabolism | Catabolism |
| 1.1.3.8 | Tryptophan utilization | Catabolism |
| 1.1.3.9 | L-cysteine catabolism | Catabolism |
| 1.1.3.10 | Lysine cleavage | Catabolism |
| 1.1.3.11 | Histidine degradation | Catabolism |
| 1.1.3.12 | Leucine degradation | Catabolism |
| 1.1.3.13 | Methionine degradation | Catabolism |
| 1.1.3.14 | Valine degradation | Catabolism |
| 1.1.3.15 | Phenylalanine, tyrosine degradation | Catabolism |
| 1.1.3.16 | Glutamine | Catabolism |
| 1.1.3.17 | Taurine | Catabolism |
| 1.1.3.18 | Arginine degradation III (agmatinase pathway) | Catabolism |
| 1.1.3.19 | Aspartate degradation | Catabolism |
| 1.1.3.20 | Isoleucine degradation | Catabolism |
| 1.1.4 | Amines | Catabolism |
| 1.1.4.1 | Phenylethylamine degradation | Catabolism |
| 1.1.4.2 | Carnitine degradation | Catabolism |
| 1.1.4.3 | Ornithine degradation | Catabolism |
| 1.1.5 | Others | Catabolism |
| 1.1.5.1 | Phenylacetic acid degradation | Catabolism |
| 1.1.5.2 | Ethanol degradation | Catabolism |
| 1.1.5.3 | Eugenol catabolism | Catabolism |
| 1.1.5.4 | Beta-ketoadipate pathway | Catabolism |
| 1.1.5.5 | Mandelate catabolism | Catabolism |
| 1.1.5.6 | (deoxy)ribose phosphate degradation | Catabolism |
| 1.1.5.7 | Formaldehyde degradation | Catabolism |
| 1.2 | Macromolecule degradation | Catabolism |
| 1.2.1 | RNA | Catabolism |
| 1.2.2 | DNA | Catabolism |
| 1.2.3 | Proteins/peptides/glycopeptides | Catabolism |
| 1.2.4 | Polysaccharides | Catabolism |
| 1.2.4.1 | Glycogen catabolism | Catabolism |
| 1.2.4.2 | Chitin catabolism | Catabolism |
| 1.3 | Energy metabolism (carbon) | Catabolism |
| 1.3.1 | Glycolysis | Catabolism |
| 1.3.2 | Pentose phosphate shunt, oxidative branch | Catabolism |
| 1.3.3 | Pyruvate dehydrogenase | Catabolism |
| 1.3.4 | Tricarboxylic acid cycle | Catabolism |
| 1.3.5 | Fermentation | Catabolism |
| 1.3.6 | Aerobic respiration | Catabolism |
| 1.3.7 | Anaerobic respiration | Catabolism |
| 1.3.8 | ATP proton motive force interconversion | Catabolism |
| 1.3.9 | Entner-Doudoroff pathway | Catabolism |
| 1.3.10 | Formate oxidation | Catabolism |
| 1.3.11 | Acyclic isoprenoid degradation | Catabolism |
| 1.4 | Energy production/transport | Other |
| 1.4.1 | Electron donor | Other |
| 1.4.2 | Electron acceptor | Other |
| 1.4.3 | Electron carrier | Other |
| 1.5 | Building block biosynthesis | Anabolism |
| 1.5.1 | Amino acids | Anabolism |
| 1.5.1.1 | Glutamate biosynthesis III | Anabolism |
| 1.5.1.2 | Glutamine | Anabolism |
| 1.5.1.3 | Arginine | Anabolism |
| 1.5.1.4 | Proline | Anabolism |
| 1.5.1.5 | Aspartate | Anabolism |
| 1.5.1.6 | Asparagine | Anabolism |
| 1.5.1.7 | Lysine, diaminopimelate | Anabolism |
| 1.5.1.8 | Threonine | Anabolism |
| 1.5.1.9 | Methionine | Anabolism |
| 1.5.1.10 | Glycine | Anabolism |
| 1.5.1.11 | Serine | Anabolism |
| 1.5.1.12 | Cysteine | Anabolism |
| 1.5.1.13 | Phenylalanine | Anabolism |
| 1.5.1.14 | Tyrosine | Anabolism |
| 1.5.1.15 | Tryptophan | Anabolism |
| 1.5.1.16 | Histidine | Anabolism |
| 1.5.1.17 | Alanine biosynthesis | Anabolism |
| 1.5.1.18 | Leucine/valine | Anabolism |
| 1.5.1.19 | Isoleucine | Anabolism |
| 1.5.1.20 | Chorismate | Anabolism |
| 1.5.1.21 | Homoserine | Anabolism |
| 1.5.1.22 | Ornithine | Anabolism |
| 1.5.1.23 | Selenocysteine | Anabolism |
| 1.5.1.24 | Glutamate biosynthesis I | Anabolism |
| 1.5.1.25 | Alanine biosynthesis III | Anabolism |
| 1.5.2 | Nucleotide | Anabolism |
| 1.5.2.1 | Purine biosynthesis | Anabolism |
| 1.5.2.2 | Pyrimidine biosynthesis | Anabolism |
| 1.5.2.3 | Purine ribonucleotide biosynthesis | Anabolism |
| 1.5.2.4 | Pyrimidine ribonucleotide/ribonucleoside biosynthesis | Anabolism |
| 1.5.3 | Cofactor, small molecule carrier | Anabolism |
| 1.5.3.1 | Biotin | Anabolism |
| 1.5.3.2 | Folic acid (tetrahydrofolate) | Anabolism |
| 1.5.3.3 | Lipoate | Anabolism |
| 1.5.3.4 | Molybdenum (molybdopterin) | Anabolism |
| 1.5.3.5 | Coenzyme A | Anabolism |
| 1.5.3.6 | Pyridoxine (vitamin B6) | Anabolism |
| 1.5.3.7 | Nicotinamide adenine dinucleotide (NAD) | Anabolism |
| 1.5.3.8 | Thiamine (Vitamin B1) | Anabolism |
| 1.5.3.9 | Riboflavin (Vitamin B2), FAD, FMN | Anabolism |
| 1.5.3.10 | Glutathione | Anabolism |
| 1.5.3.11 | Menaquinone (MK), ubiquinone (Q) | Anabolism |
| 1.5.3.12 | Heme, porphyrin | Anabolism |
| 1.5.3.13 | Cobalamin (Vitamin B12) | Anabolism |
| 1.5.3.14 | Enterochelin (enterobactin) | Anabolism |
| 1.5.3.15 | Chrysobactin | Anabolism |
| 1.5.3.16 | Achromobactin | Anabolism |
| 1.5.3.19 | Isoprenoid biosynthesis | Anabolism |
| 1.5.3.20 | Tetrahydrobiopterin biosynthesis | Anabolism |
| 1.5.3.21 | Pyochelin biosynthesis | Anabolism |
| 1.5.3.22 | Glutathione redox reactions | Other |
| 1.5.3.23 | Pantothenate biosynthesis I | Anabolism |
| 1.5.3.24 | Folate polyglutamylation | Other |
| 1.5.4 | Fatty acid and phosphatidic acid | Anabolism |
| 1.5.4.1 | Fatty acid elongation - unsaturated | Other |
| 1.5.4.2 | Fatty acid elongation - saturated | Other |
| 1.5.4.3 | Fatty acid biosynthesis -- initial steps | Other |
| 1.6 | Macromolecules (cellular constituent) biosynthesis | Anabolism |
| 1.6.1 | Phospholipid | Anabolism |
| 1.6.1.1 | Cyclopropane fatty acid (CFA) biosynthesis | Anabolism |
| 1.6.1.2 | Phospholipid biosynthesis I | Anabolism |
| 1.6.2 | Colanic acid (M antigen) | Anabolism |
| 1.6.3 | Lipopolysaccharide | Anabolism |
| 1.6.3.1 | O antigen | Anabolism |
| 1.6.3.2 | Core region | Anabolism |
| 1.6.3.3 | Lipid A | Anabolism |
| 1.6.4 | Enterobacterial common antigen (surface glycolipid) | Anabolism |
| 1.6.5 | K antigen | Anabolism |
| 1.6.6 | Osmoregulated periplasmic glucan | Anabolism |
| 1.6.7 | Peptidoglycan (murein) | Anabolism |
| 1.6.9 | Polysaccharides, cytoplasmic | Anabolism |
| 1.6.10 | Lipoprotein | Anabolism |
| 1.6.11 | Glycoprotein | Anabolism |
| 1.6.12 | Flagella | Anabolism |
| 1.6.13 | Fimbria, pili, curli | Anabolism |
| 1.6.15 | Large molecule carriers | Anabolism |
| 1.6.15.1 | Cytochromes | Anabolism |
| 1.6.15.2 | Thioredoxin, glutaredoxin | Anabolism |
| 1.6.15.3 | Biotin carboxyl carrier protein | Anabolism |
| 1.6.15.4 | Acyl carrier protein | Anabolism |
| 1.6.16 | Cellulose biosynthesis | Anabolism |
| 1.6.17 | GDP-mannose metabolism | Anabolism |
| 1.6.18 | Siderophore | Anabolism |
| 1.7 | Central intermediary metabolism | Other |
| 1.7.1 | Unassigned reversible reactions | Other |
| 1.7.2 | Glyoxylate bypass | Anabolism |
| 1.7.3 | Pentose phosphate shunt, non-oxidative branch | Other |
| 1.7.6 | Glycerol metabolism | Catabolism |
| 1.7.6.1 | Glycerol degradation I | Catabolism |
| 1.7.6.2 | Glycerol degradation II | Catabolism |
| 1.7.7 | Galactose metabolism | Other |
| 1.7.8 | Gluconeogenesis | Anabolism |
| 1.7.9 | Misc. glucose metabolism | Other |
| 1.7.10 | Sugar nucleotide biosynthesis, conversions | Anabolism |
| 1.7.12 | Amino sugar conversions | Anabolism |
| 1.7.13 | Amino acid conversion | Other |
| 1.7.14 | Polyamine biosynthesis | Anabolism |
| 1.7.14.1 | Putrescine biosynthesis I | Anabolism |
| 1.7.14.2 | Putrescine biosynthesis III | Anabolism |
| 1.7.14.3 | Spermidine biosynthesis | Anabolism |
| 1.7.15 | 2'-deoxyribonucleotide/ribonucleoside metabolism | Anabolism |
| 1.7.17 | Formyl-tetrahydrofolate biosynthesis | Anabolism |
| 1.7.18 | Betaine biosynthesis | Anabolism |
| 1.7.19 | Incorporation of metal ions | Other |
| 1.7.20 | S-adenosyl methionine biosynthesis | Anabolism |
| 1.7.21 | Glyoxylate degradation | Catabolism |
| 1.7.22 | Carnitine metabolism | Catabolism |
| 1.7.23 | Methylglyoxal metabolism | Other |
| 1.7.24 | Cyanate catabolism | Catabolism |
| 1.7.25 | Glycolate metabolism | Catabolism |
| 1.7.26 | Allantoin assimilation | Catabolism |
| 1.7.27 | Pyridoxal 5'-phosphate salvage | Other |
| 1.7.28 | Pyruvate catabolism | Catabolism |
| 1.7.29 | Acetate catabolism | Catabolism |
| 1.7.31 | Aminobutyrate catabolism | Catabolism |
| 1.7.32 | Putrescine catabolism | Catabolism |
| 1.7.32.1 | Putrescine degradation I | Catabolism |
| 1.7.32.2 | Putrescine degradation II | Catabolism |
| 1.7.33 | Nucleotide and nucleoside conversions | Other |
| 1.7.33.1 | Salvage pathways of adenine, hypoxanthine, and their nucleosides | Other |
| 1.7.33.2 | Salvage pathways of guanine, xanthine, and their nucleosides | Other |
| 1.7.33.3 | Salvage pathways of pyrimidine deoxyribonucleotides | Other |
| 1.7.33.4 | Salvage pathways of pyrimidine ribonucleotides | Other |
| 1.7.34 | Peptidoglycan (murein) turnover, recycling | Other |
| 1.7.35 | Lactate oxidation | Other |
| 1.7.36 | Trehalose biosynthesis | Anabolism |
| 1.7.37 | C1 assimilation, serine pathway | Catabolism |
| 1.7.38 | Methionine salvage pathway | Other |
| 1.7.39 | Conversion of succinate to propionate | Other |
| 1.7.40 | Fructoselysine and psicoselysine degradation | Catabolism |
| 1.7.41 | PRPP biosynthesis I | Anabolism |
| 1.7.42 | PRPP biosynthesis II | Anabolism |
| 1.7.43 | ppGpp biosynthesis | Anabolism |
| 1.7.44 | NAD phosphorylation and dephosphorylation | Other |
| 1.7.45 | Pyridine nucleotide cycling | Other |
| 1.7.46 | Pyridine nucleotide synthesis | Anabolism |
| 1.8 | Metabolism of other compounds | Other |
| 1.8.1 | Phosphorous metabolism | Other |
| 1.8.2 | Sulfur metabolism | Other |
| 1.8.2.1 | Sulfate assimilation | Catabolism |
| 1.8.3 | Nitrogen metabolism | Other |
| 2 | Information transfer | Other |
| 2.1 | DNA related | Other |
| 2.1.1 | DNA replication | Other |
| 2.1.2 | DNA restriction/modification | Other |
| 2.1.3 | DNA recombination | Other |
| 2.1.4 | DNA repair | Other |
| 2.1.5 | DNA degradation | Catabolism |
| 2.2 | RNA related | Other |
| 2.2.2 | Transcription related | Other |
| 2.2.3 | RNA modification | Other |
| 2.2.4 | RNA degradation | Catabolism |
| 2.2.5 | tRNA | Other |
| 2.2.6 | rRNA, stable RNA | Other |
| 2.2.7 | Antisense RNA | Other |
| 2.3 | Protein related | Anabolism |
| 2.3.1 | Amino acid-activation | Other |
| 2.3.2 | Translation | Anabolism |
| 2.3.3 | Posttranslational modification | Other |
| 2.3.4 | Chaperoning, folding | Other |
| 2.3.5 | Export, signal peptide cleavage | Transport |
| 2.3.6 | Turnover, degradation | Catabolism |
| 2.3.7 | Nucleoproteins, basic proteins | Other |
| 2.3.8 | Ribosomal proteins | Other |
| 2.3.9 | Non-ribosomal peptide synthetase | Anabolism |
| 3 | Regulation | Other |
| 3.1. | Type of regulation | Other |
| 3.1.1 | DNA structure level | Other |
| 3.1.1.1 | DNA bending, supercoiling, inversion | Other |
| 3.1.1.2 | Methylation | Other |
| 3.1.2 | Transcriptional level | Other |
| 3.1.2.1 | Sigma factors, anti-sigmafactors | Other |
| 3.1.2.2 | Activator | Other |
| 3.1.2.3 | Repressor | Other |
| 3.1.2.4 | Complex regulation | Other |
| 3.1.2.4.1 | More than one signal needed | Other |
| 3.1.2.4.2 | Regulons or multilayer component regulatory systems | Other |
| 3.1.2.4.3 | Two-component regulatory systems (external signal) | Other |
| 3.1.2.4.4 | Quorum sensing | Other |
| 3.1.2.5 | Action unknown | Other |
| 3.1.3 | Posttranscriptional | Other |
| 3.1.3.1 | Translation attenuation and efficiency | Other |
| 3.1.3.2 | Covalent modification, demodification, maturation | Other |
| 3.1.3.3 | Inhibition / activation of enzymes | Other |
| 3.1.3.4 | Proteases, cleavage of compounds | Other |
| 3.1.3.5 | Multilayer regulatory systems | Other |
| 3.1.3.6 | Antisense RNA | Other |
| 3.1.3.7 | binding, sequestering | Other |
| 3.1.4 | Regulation level unknown | Other |
| 3.3 | Genetic unit regulated | Other |
| 3.3.1 | Operon (regulation of one operon) | Other |
| 3.3.2 | Regulon (a network of operons encoding related functions) | Other |
| 3.3.3 | Stimulon (ie. environmental stimulus) | Other |
| 3.3.4 | Global | Other |
| 3.4 | Trigger (some information added) | Other |
| 3.5 | Trigger modulation (some information added) | Other |
| 4 | Transport | Transport |
| 4.1 | Channel-type Transporters | Transport |
| 4.1.A. | alpha-type channels | Transport |
| 4.1.A.1. | The Voltage-gated Ion Channel (VIC) Superfamily | Transport |
| 4.1.A.8. | The Major Intrinsic Protein (MIP) Family | Transport |
| 4.1.A.11 | The Chloride Channel (ClC) Family | Transport |
| 4.1.B.17 | The Outer Membrane Factor (OMF) Family | Transport |
| 4.1.A.22 | The Large Conductance Mechanosensitive Ion Channel (MscL) Family | Transport |
| 4.1.A.23 | The Small Conductance Mechanosensitive Ion Channel (MscS) Family | Transport |
| 4.1.A.46 | The H+- or Na+-translocating Bacterial Flagellar Motor (Mot) | Transport |
| 4.1.B | Beta barrel porins (The Outer Membrane Porin (OMP) Functional | Transport |
| 4.1.B.1 | The General Bacterial Porin (GBP) Family | Transport |
| 4.1.B.3 | The Sugar Porin (SP) Family | Transport |
| 4.1.B.9 | The FadL Outer Membrane Protein (FadL) Family | Transport |
| 4.1.B.10 | The Nucleoside-specific Channel-forming Outer Membrane Porin (Tsx) Family | Transport |
| 4.1.B.14 | The Outer Membrane Receptor (OMR) Family | Transport |
| 4.1.B.21 | The OmpG Porin (OmpG) Family | Transport |
| 4.2 | Electrochemical potential driven transporters | Transport |
| 4.2.A. | Porters (Uni-, Sym- and Antiporters) | Transport |
| 4.2.A.1 | The Major Facilitator Superfamily (MFS) | Transport |
| 4.2.A.2 | The Glycoside-Pentoside-Hexuronide (GPH):Cation Symporter Family | Transport |
| 4.2.A.3 | The Amino Acid-Polyamine-Choline (APC) Family | Transport |
| 4.2.A.4 | The Cation Diffusion Facilitator (CDF) Family | Transport |
| 4.2.A.6 | The Resistance-Nodulation-Cell Division (RND) Superfamily | Transport |
| 4.2.A.7 | The Drug/Metabolite Transporter (DMT) Superfamily | Transport |
| 4.2.A.8 | The Gluconate:H+ Symporter (GntP) Family | Transport |
| 4.2.A.9 | The Cytochrome oxidase biogenesis (Oxa1) Family | Transport |
| 4.2.A.10 | The 2-Keto-3-Deoxygluconate Transporter (KDGT) Family | Transport |
| 4.2.A.13 | The C4-Dicarboxylate Uptake (Dcu) Family | Transport |
| 4.2.A.14 | The Lactate Permease (LctP) Family | Transport |
| 4.2.A.15 | The Betaine/Carnitine/Choline Transporter (BCCT) Family | Transport |
| 4.2.A.16 | The Telurite-resistance/Dicarboxylate Transporter (TDT) Family | Transport |
| 4.2.A.17 | The Proton-dependent Oligopeptide Transporter (POT) Family | Transport |
| 4.2.A.19 | The Ca2+:Cation Antiporter (CaCA) Family | Transport |
| 4.2.A.20 | The Inorganic Phosphate Transporter (PiT) Family | Transport |
| 4.2.A.21 | The Solute:Sodium Symporter (SSS) Family | Transport |
| 4.2.A.23 | The Dicarboxylate/Amino Acid:Cation (Na+ or H+) Symporter (DAACS) Family | Transport |
| 4.2.A.25 | The Alanine/Glycine:Cation symporter (AGCS) Family | Transport |
| 4.2.A.26 | The Branched Chain Amino Acid:Cation Symporter (LIVCS) Family | Transport |
| 4.2.A.27 | The Glutamate:Na+ Symporter (GltS) Family | Transport |
| 4.2.A.33 | The NhaA Na+:H+ Antiporter (NhaA) Family | Transport |
| 4.2.A.34 | The NhaB Na+:H+ Antiporter (NhaB) Family | Transport |
| 4.2.A.36 | The Monovalent Cation:Proton Antiporter-1 (CPA1) Family | Transport |
| 4.2.A.37 | The Monovalent Cation:Proton Antiporter-2 (CPA2) Family | Transport |
| 4.2.A.38 | The K+ Transporter (Trk) Family | Transport |
| 4.2.A.39 | The Nucleobase:Cation Symporter-1 (NCS1) Family | Transport |
| 4.2.A.40 | The Nucleobase:Cation Symporter-2 (NCS2) Family | Transport |
| 4.2.A.41 | The Concentrative Nucleoside Transporter (CNT) Family | Transport |
| 4.2.A.42 | The Hydroxy/Aromatic Amino Acid Permease (HAAAP) Family | Transport |
| 4.2.A.44 | The Formate-Nitrite Transporter (FNT) Family | Transport |
| 4.2.A.45 | The Metal Ion Transporter (MIT) Family | Transport |
| 4.2.A.47 | The Divalent Anion:Na+ Symporter (DASS) Family | Transport |
| 4.2.A.49 | The Ammonium Transporter (Amt) Family | Transport |
| 4.2.A.53 | The Sulfate Permease (SulP) Family | Transport |
| 4.2.A.55 | The Manganese (Nramp) Fam. | Transport |
| 4.2.A.58 | The Phosphate:Na+ Symporter (PNaS) Family | Transport |
| 4.2.A.61 | The C4-dicarboxylate Uptake C (DcuC) Family | Transport |
| 4.2.A.64 | The Type V Secretory Pathway or Twin Arginine Targeting | Transport |
| 4.2.A.66 | The Multi Antimicrobial Extrusion (MATE) Family | Transport |
| 4.2.A.68 | The p-Aminobenzoyl-glutamate Transporter (AbgT) Family | Transport |
| 4.2.A.69 | The Auxin Efflux Carrier (AEC) Family | Transport |
| 4.2.A.72 | The K+ uptake permease (KUP) family | Transport |
| 4.2.A.75 | The L-lysine exporter (LysE) family | Transport |
| 4.2.A.76 | The Resistance to Homoserine/Threonine (RhtB) Family | Transport |
| 4.2.C | Ion-gradient driven energizers | Transport |
| 4.2.C.1 | TonB Family of Auxiliary Proteins for Energization of OMR-mediated | Transport |
| 4.3 | Primary Active Transporters | Transport |
| 4.3.A. | Pyrophosphate Bond (ATP; GTP; P2) Hydrolysis-driven Active Transporters | Transport |
| 4.3.A.1 | The ATP-binding Cassette (ABC) Superfamily + ABC-type Uptake Permeases | Transport |
| 4.3.A.1.a | ATP binding component | Transport |
| 4.3.A.1.m | membrane component | Transport |
| 4.3.A.1.p | periplasmic binding component | Transport |
| 4.3.A.1.am | ATP binding and membrane component | Transport |
| 4.3.A.2 | The H+/Na+-translocating F-, V- and A-type ATPase (F-ATPase) Superfamily | Transport |
| 4.3.A.3 | The P-type ATPase (P-ATPase) Superfamily | Transport |
| 4.3.A.4 | The Arsenite-Antimonite (Ars) Efflux Family | Transport |
| 4.3.A.5 | The Type II (General) Secretory Pathway (IISP) Family | Transport |
| 4.3.A.6 | The Type III (Virulence-related) Secretory Pathway (IIISP) Family | Transport |
| 4.3.A.7 | The Type IV (Conjugal DNA-Protein Transfer) Secretory Pathway (IVSP) | Transport |
| 4.3.D. | Oxidoreduction-driven Active Transporters | Transport |
| 4.3.D.1 | The Proton- or sodium ion-translocating NADH Dehydrogenase (NDH) Family | Transport |
| 4.3.D.4 | The Proton-translocating Cytochrome Oxidase (COX) Superfamily | Transport |
| 4.4 | Group Translocators | Transport |
| 4.4.A | Phosphotransferase Systems (PEP-dependent PTS) | Transport |
| 4.4.A.1 | The PTS Glucose-Glucoside (Glc) Family | Transport |
| 4.4.A.2 | The PTS Fructose-Mannitol (Fru) Family | Transport |
| 4.4.A.3 | The PTS Lactose-N,N'-Diacetylchitobiose-betaucoside (Lac) Family | Transport |
| 4.4.A.4 | The PTS Glucitol (Gut) Family | Transport |
| 4.4.A.5 | The PTS Galactitol (Gat) Family | Transport |
| 4.4.A.6 | The PTS Mannose-Fructose-Sorbose (Man) Family | Transport |
| 4.8.A | Accessory Factors Involved in Transport | Transport |
| 4.8.A.1 | The Membrane Fusion Protein (MFP) Family | Transport |
| 4.8.A.2 | The Secretin Auxiliary Lipoprotein (SAL) Family | Transport |
| 4.8.A.3 | MPA1 Family auxillary transport protein | Transport |
| 4.8.A.7 | The Phosphotransferase System Enzyme I (EI) Family | Transport |
| 4.8.A.8 | The Phosphotransferase System HPr (HPr) Family | Transport |
| 4.9.A | Transporters of Unknown Classification | Transport |
| 4.9.A | Recognized transporters of unknown biochemical mechanism | Transport |
| 4.9.A.1 | The Polysaccharide Transporter (PST) Family | Transport |
| 4.9.A.4 | The Nicotinamide Mononucleotide (NMN) Uptake Permease (PnuC) Family | Transport |
| 4.9.A.8 | The Ferrous Iron Uptake (FeoB) Family | Transport |
| 4.9.A.13 | The Short Chain Fatty Acid Transporter (scFAT) Family | Transport |
| 4.9.A.16 | The Septal DNA Translocator (SDT) Family | Transport |
| 4.9.A.17 | The Metal Ion Transporter (MIT) Family | Transport |
| 4.9.B | Putative uncharacterized transport protein | Transport |
| 4.9.B.3 | The Putative Bacterial Murein Precursor Exporter (MPE) Family | Transport |
| 4.9.B.4 | The Putative Efflux Transporter (PET) Family | Transport |
| 4.9.B.6 | The Toxic Hok/Gef Protein (Hok/Gef) Family | Transport |
| 4.9.B.10 | The 6TMS Putative MarC Transporter (MarC) Family | Transport |
| 4.9.B.18 | The SecDF-associated Single Transmembrane Protein (SSTP) Family | Transport |
| 4.9.B.21 | The Frataxin (Frataxin) Family | Transport |
| 4.9.B.22 | The Putative Permease (PerM) Family | Transport |
| 4.9.B.24 | The Testis-Enhanced Gene Transfer (TEGT) Family | Transport |
| 4.9.B.25 | The YbbM (YbbM) Family | Transport |
| 4.9.B.27 | The YdjX-Z (YdjX-Z) Family | Transport |
| 4.9.B.28 | The YqaE (YqaE) Family | Transport |
| 4.9.B.29 | The YebN (YebN) Family | Transport |
| 4.9.B.31 | The YqiH (YqiH) Family | Transport |
| 4.9.B.32 | The Putative Vectorial Glycosyl Polymerization (VGP) Family | Transport |
| 4.S | substrate | Transport |
| 4.S.1 | (D)-galactarate | Transport |
| 4.S.2 | 2-keto-3-deoxy-D-gluconate | Transport |
| 4.S.3 | 3-hydroxyphenylpropionic acid | Transport |
| 4.S.4 | 3-phenylpropionic acid | Transport |
| 4.S.5 | alkanesulfonate | Transport |
| 4.S.6 | alkanesulphonate | Transport |
| 4.S.8 | alkylphosphonate | Transport |
| 4.S.9 | allantoin | Transport |
| 4.S.10 | allose/ribose | Transport |
| 4.S.11 | alpha-ketoglutarate | Transport |
| 4.S.12 | amino acid | Transport |
| 4.S.13 | amino acid/amide | Transport |
| 4.S.14 | ammonium | Transport |
| 4.S.15 | antibiotic | Transport |
| 4.S.16 | arabinose polymer | Transport |
| 4.S.17 | arginine | Transport |
| 4.S.18 | arginine/ornithine | Transport |
| 4.S.19 | arsenite | Transport |
| 4.S.20 | benzoate | Transport |
| 4.S.21 | lactose/glucose | Transport |
| 4.S.22 | beta-glucoside | Transport |
| 4.S.25 | Ca+/ H+ | Transport |
| 4.S.26 | cadaverine/lysine | Transport |
| 4.S.27 | carnitine | Transport |
| 4.S.28 | cation | Transport |
| 4.S.29 | cellobiose/arbutin/salicin | Transport |
| 4.S.31 | chloramphenicol | Transport |
| 4.S.32 | chloride | Transport |
| 4.S.33 | choline | Transport |
| 4.S.34 | citrate/succinate | Transport |
| 4.S.35 | colicin | Transport |
| 4.S.36 | Cu+ | Transport |
| 4.S.37 | curli subunit | Transport |
| 4.S.38 | cyanate | Transport |
| 4.S.39 | cysteine | Transport |
| 4.S.40 | cysteine/O-acetyl-L-serine/cysteine metabolites | Transport |
| 4.S.41 | cytosine | Transport |
| 4.S.42 | D-alanine/D-serine/glycine | Transport |
| 4.S.43 | D-galactonate | Transport |
| 4.S.44 | D-glucarate | Transport |
| 4.S.45 | D-glucose/trehalose | Transport |
| 4.S.46 | dicarboxylate | Transport |
| 4.S.47 | dipeptide | Transport |
| 4.S.48 | D-ribose | Transport |
| 4.S.49 | drug | Transport |
| 4.S.50 | D-xylose | Transport |
| 4.S.51 | enterochelin | Transport |
| 4.S.52 | fatty acid | Transport |
| 4.S.53 | Fe | Transport |
| 4.S.54 | Fe++ | Transport |
| 4.S.56 | ferric enterobactin | Transport |
| 4.S.57 | ferric hydroxamate | Transport |
| 4.S.58 | ferrichrome | Transport |
| 4.S.59 | formate | Transport |
| 4.S.60 | formate/oxalate | Transport |
| 4.S.61 | fosfidomycin/H+ | Transport |
| 4.S.63 | fructose | Transport |
| 4.S.64 | fucose | Transport |
| 4.S.65 | galactitol | Transport |
| 4.S.66 | galactose/H+ | Transport |
| 4.S.67 | gamma-aminobutyrate | Transport |
| 4.S.68 | glucitol/sorbitol | Transport |
| 4.S.69 | gluconate | Transport |
| 4.S.70 | gluconate/L-idonate | Transport |
| 4.S.71 | glucose | Transport |
| 4.S.72 | glucose/maltose | Transport |
| 4.S.73 | glucuronide | Transport |
| 4.S.74 | glutamate/aminobutyric acid | Transport |
| 4.S.75 | glutamate/aspartate | Transport |
| 4.S.76 | glutamine | Transport |
| 4.S.77 | glycerol | Transport |
| 4.S.78 | glycerol-3-P | Transport |
| 4.S.79 | glycine betaine/choline | Transport |
| 4.S.80 | glycine betaine/proline | Transport |
| 4.S.81 | group A colicin | Transport |
| 4.S.82 | H+ | Transport |
| 4.S.83 | H+/acridine | Transport |
| 4.S.84 | H+/lactose/glucose | Transport |
| 4.S.85 | heme | Transport |
| 4.S.86 | hexose phosphate | Transport |
| 4.S.87 | hexuronate | Transport |
| 4.S.88 | histidine | Transport |
| 4.S.89 | histidine/lysine/arginine/ornithine | Transport |
| 4.S.90 | homoserine/lactone | Transport |
| 4.S.91 | hydrophilic molecule | Transport |
| 4.S.92 | hydrophilic molecules | Transport |
| 4.S.93 | ion | Transport |
| 4.S.95 | iron dicitrate | Transport |
| 4.S.96 | K+ | Transport |
| 4.S.97 | K+/H+ | Transport |
| 4.S.98 | lactate | Transport |
| 4.S.99 | lactose | Transport |
| 4.S.100 | L-arabinose | Transport |
| 4.S.101 | L-arabinose /H+ | Transport |
| 4.S.102 | L-arabinose/ isopropyl-beta-D-thiogalactopyranoside | Transport |
| 4.S.103 | L-asparagine | Transport |
| 4.S.104 | lipooligosaccharides | Transport |
| 4.S.105 | lipopolysaccharide | Transport |
| 4.S.106 | lipoprotein | Transport |
| 4.S.107 | L-lactate | Transport |
| 4.S.108 | L-leucine/L-valine/L-iso-leucine | Transport |
| 4.S.109 | L-rhamnose/H+ | Transport |
| 4.S.110 | L-threonine/L-serine | Transport |
| 4.S.111 | lysine | Transport |
| 4.S.112 | lysine/arginine/ornithine | Transport |
| 4.S.113 | maltose | Transport |
| 4.S.114 | maltose/maltodextrin | Transport |
| 4.S.115 | mannitol | Transport |
| 4.S.116 | mannose | Transport |
| 4.S.117 | melibiose | Transport |
| 4.S.118 | methionine | Transport |
| 4.S.119 | methylgalactoside/galactose | Transport |
| 4.S.120 | Mg++ | Transport |
| 4.S.121 | Mg2+/Ni2+/Co2+ | Transport |
| 4.S.122 | microcin B17 | Transport |
| 4.S.123 | Mn+/H+ | Transport |
| 4.S.124 | molybdate | Transport |
| 4.S.125 | molybdenum | Transport |
| 4.S.126 | multidrug | Transport |
| 4.S.127 | multidrug/bicyclomycin | Transport |
| 4.S.128 | muropeptide | Transport |
| 4.S.129 | myo-inositol | Transport |
| 4.S.130 | Na+ | Transport |
| 4.S.131 | Na+/ alanine/glycine | Transport |
| 4.S.132 | Na+/ H+ | Transport |
| 4.S.133 | Na+/Ca+ | Transport |
| 4.S.134 | Na+/dicarboxylate | Transport |
| 4.S.135 | Na+/glutamate/aspartate | Transport |
| 4.S.136 | Na+/H+ | Transport |
| 4.S.137 | Na+/leucine/valine/iso-leucine | Transport |
| 4.S.138 | Na+/pantothenate | Transport |
| 4.S.139 | Na+/proline | Transport |
| 4.S.140 | Na+/serine/threonine | Transport |
| 4.S.141 | N-acetylgalactosamine | Transport |
| 4.S.142 | N-acetylglucosamine | Transport |
| 4.S.143 | Ni++ | Transport |
| 4.S.144 | nicotinamide mononucleotide | Transport |
| 4.S.145 | nitrite | Transport |
| 4.S.146 | nucleoside | Transport |
| 4.S.147 | nucleoside/H+ | Transport |
| 4.S.148 | oligopeptide | Transport |
| 4.S.150 | p-aminobenzoyl-glutamate | Transport |
| 4.S.151 | Pb/Cd/Zn/Hg | Transport |
| 4.S.152 | peptide | Transport |
| 4.S.153 | phenylalanine | Transport |
| 4.S.154 | phenylalanine/ tyrosine | Transport |
| 4.S.155 | phosphate | Transport |
| 4.S.156 | polymyxin | Transport |
| 4.S.158 | proline | Transport |
| 4.S.159 | proline/betaine | Transport |
| 4.S.160 | protein | Transport |
| 4.S.161 | protein/DNA | Transport |
| 4.S.162 | purine/xanthine | Transport |
| 4.S.163 | putrescine | Transport |
| 4.S.164 | putrescine/ornithine | Transport |
| 4.S.166 | putrescine/spermidine | Transport |
| 4.S.167 | serine | Transport |
| 4.S.168 | shikimate/dehydroshikimate | Transport |
| 4.S.169 | sialic acid | Transport |
| 4.S.170 | S-methylmethionine | Transport |
| 4.S.172 | sucrose | Transport |
| 4.S.173 | sugar | Transport |
| 4.S.174 | sulfate | Transport |
| 4.S.175 | taurine | Transport |
| 4.S.176 | tellurite | Transport |
| 4.S.177 | thiamine | Transport |
| 4.S.178 | thiosulfate | Transport |
| 4.S.179 | thiosulfate/sulfate | Transport |
| 4.S.180 | threonine | Transport |
| 4.S.181 | trehalose | Transport |
| 4.S.182 | tripeptide | Transport |
| 4.S.183 | tryptophan | Transport |
| 4.S.184 | Tyrosine | Transport |
| 4.S.185 | uracil | Transport |
| 4.S.187 | vitamin B12 | Transport |
| 4.S.188 | water | Transport |
| 4.S.189 | xanthosine | Transport |
| 4.S.190 | xylose/H+ | Transport |
| 4.S.191 | Zn | Transport |
| 4.S.192 | chrysobactin | Transport |
| 4.S.193 | achromobactin | Transport |
| 4.S.194 | acetate | Transport |
| 4.S.195 | 2-O-alpha-mannosyl-D-glycerate | Transport |
| 4.S.196 | Zn(2+) | Transport |
| 4.S.197 | N-acetylmuramic acid (MurNAc) | Transport |
| 5 | Cell processes | Other |
| 5.1 | Cell division | Other |
| 5.2 | Cell cycle physiology | Other |
| 5.3 | Motility (incl. chemotaxis, energytaxis, aerotaxis, redoxtaxis) | Other |
| 5.4 | Genetic exchange, recombination | Other |
| 5.5 | Adaptation to stress | Other |
| 5.5.1 | Osmotic pressure | Other |
| 5.5.2 | Temperature extremes | Other |
| 5.5.3 | Starvation response | Other |
| 5.5.4 | pH response | Other |
| 5.5.5 | Dessication | Other |
| 5.5.6 | Other stresses (mechanical, nutritional, oxidative) | Other |
| 5.5.7 | Fe aquisition | Other |
| 5.6 | Protection | Other |
| 5.6.1 | Radiation | Other |
| 5.6.2 | Detoxification (xenobiotic metabolism) | Other |
| 5.6.3 | Cell killing | Other |
| 5.6.4 | Drug resistance/sensitivity | Other |
| 5.8 | SOS response | Other |
| 5.1 | Defense/survival | Other |
| 5.11 | DNA uptake | Other |
| 5.12 | Biofilm production | Other |
| 5.13 | Virulence associated | Other |
| 5.14 | Cell-cell communication | Other |
| 6 | Cell structure | Other |
| 6.1 | Membrane | Other |
| 6.2 | Peptidoglycan (murein) | Other |
| 6.3 | Surface antigens (ECA, O antigen of LPS) | Other |
| 6.4 | Flagellum | Other |
| 6.5 | Pilus | Other |
| 6.6 | Ribosome | Other |
| 6.7 | Capsule (M and K antigens) | Other |
| 7 | Location of gene products | Other |
| 7.1 | Cytoplasm | Other |
| 7.2 | Periplasmic space | Other |
| 7.3 | Inner membrane | Other |
| 7.4 | Outer membrane | Other |
| 7.5 | Extracellular | Other |
| 8 | extrachromosomal | Other |
| 8.1 | Prophage genes and phage related functions | Other |
| 8.1.1 | DNA packaging, phage assembly | Other |
| 8.1.2 | Replication | Other |
| 8.1.3 | Regulation | Other |
| 8.1.4 | Integration, recombination | Other |
| 8.1.5 | Lysis | Other |
| 8.1.6 | Structural component | Other |
| 8.2 | Plasmid related | Other |
| 8.2.1 | replication and maintenance | Other |
| 8.2.2 | plasmid transfer | Other |
| 8.3 | Transposon related | Other |
| 8.3.1 | transposases | Other |
| 8.3.2 | regulation of mobility | Other |
| 8.4 | Colicin related | Other |
| 9 | DNA sites | Other |
| 10 | cryptic genes | Other |
