# Supplementary material for: Transcription Factors in Escherichia coli Prefer the Holo Conformation
Source: PLoS One. 2013 Jun 12;8(6):e65723. doi: 10.1371/journal.pone.0065723 (PMC3680503; doi:10.1371/journal.pone.0065723)
Supplement: Table S4 — Transcriptional regulation in amino acid pathways. Regulation of amino acid pathways by TFs and by attenuation. (DOCX) [file pone.0065723.s017.docx]

**Table S4. Transcriptional regulation in amino acid pathways**

| **Amino acid** | **TFs - function - conformation in all the genes that belong to the biosynthetic pathway** | **Attenuation** | **Demand in the colon** |
| --- | --- | --- | --- |
| Alanine | [SgrR + no effector] | No | High |
| Argininine | [ArgR - holo] | No | Low |
| Asparagine | [AsnC ± apo] | No | High |
| Aspartate | [DcuR + holo] [CRP + holo] [NarL - holo] | No | High |
| Cysteine | [CysB ± holo] [Cbl + apo] [ IHF ± no effector] | No | High |
| Glutamate | [FlhDC + no effector] [GadX + no effector] [AdiY + no effector] [GadE + no effector] [GadW + no effector] [ArgP + holo] [CRP + holo] [IHF + no effector] [Lrp +] [HdfR ± no effector] | No, but it has a small RNA (SroC) | Low |
| Glutamine | [Fis + no effector] [Crp - holo] [IHF + no effector] [NtrC ± holo] | No, but there is a terminator in the gene *glnL* | Low |
| Glicine | [Lrp ±] [PurR - holo] [MetR -] [CRP - holo] | No | ? |
| Histidine | No TFs | Yes | High |
| Isoleucine | [Lrp ±] [IHF ± no effector] [Crp + holo] [IlvY +] | Yes | High |
| Valine | [Lrp ±] [IHF ± no effector] [Crp + holo] [IlvY +] | Yes | High |
| Leucine | [Lrp ±] [IHF ± no effector] [Crp + holo] [IlvY +] [TyrR + holo] | Yes | High |
| Lysine | [ArgP + holo] [DksA +] | Yes | Low |
| Methionine | [ArgP + holo] [DksA +] [MetJ - holo] [MalI -] [CRP + holo] [FadR ± ] | Yes | High |
| Phenylalanine | [TyrR - holo] [CpxR + holo] [TrpR - holo] [Crp - holo] [Lrp +] [IHF +- no effector] | Yes | High |
| Proline | [Fis + no effector] [Crp ± holo] [H-NS -][PhoB +-] | No | High |
| Serine | [Lrp ±] [CRP - holo] | No | High |
| Threonine | [ArgP + holo] [DksA +] | Yes | High |
| Tryptophan | [TyrR - holo] [CpxR + holo] [TrpR - holo] [Crp - holo] [Lrp +] | Yes | Low |
| Tyrosine | [TyrR - holo] [CpxR + holo] [TrpR - holo] [Crp - holo] [Lrp +] | No | Low |
