# Supplementary material for: Transcription Factors in Escherichia coli Prefer the Holo Conformation
Source: PLoS One. 2013 Jun 12;8(6):e65723. doi: 10.1371/journal.pone.0065723 (PMC3680503; doi:10.1371/journal.pone.0065723)
Supplement: Table S5 — Prediction of attenuators in genes that belong to amino acid biosynthesis pathways. Predictions obtained from Merino & Yanofsky (2005). (DOC) [file pone.0065723.s018.doc]

**Table S5**. Predictions of attenuators in genes that belong to amino acid biosynthesis pathways

| **Gene** | **Purpose** | **Direction** | **Sequence** |
| --- | --- | --- | --- |
| *asnA* | Translational | Forward | [terminator,3925124,3925171,-116,gugugucgguUUUUGUUGCUUAAUCAUAAGCAACAGGAcgcaggagua] |
| *asnS* | Transcriptional | Reverse | [terminator,988333,988379,-129,uaauaucaucAGGGAGCUAAUCGGCUCCCUUUUUUUAccuuuaauuc] [anti-terminator,988354,988413,-54,agcguucgacCUUCCGCAUAUUAAAAAAGCCAGUUAAUAUCAUCAGGGAGcuaaucggcu] [anti-anti-terminator,988381,988453,-168,cuaucugccaUGAUAAAGCGUUUGUUCGGGCGCUGCGCAAAGCGUUCGACCUUCCGCAUAUUAaaaaagccag] |
| *glnH* | Transcriptional | Reverse | [terminator,847320,847385,-97,ccccagaaugGUGCAUCUUCAGGGUAUUGCCCUAUAAAUCGUGCAUCACGUUUUUGccgcaucucg] [anti-terminator,847362,847409,-13,caauuuuagcGCACCAGAUUGGUGCCCCAGAAUGGUGCaucuucaggg] [anti-anti-terminator,847375,847409,-72,caauuuuagcGCACCAGAUUGGUGCcccagaaugg] |
| *thrA* | Transcriptional | Forward | [anti-anti-terminator,190,265,-145,augaaacgcaUUAGCACCACCAUUACCACCACCAUCACCAUUACCACAGGUAACGGUGCGGGCUGAcgcguacagg] [anti-terminator,234,298,-1941,cacagguaacGGUGCGGGCUGACGCGUACAGGAAACACAGAAAAAAGCCCGCACCugacagugcg] [terminator,266,321,-20,aaacacagaaAAAAGCCCGCACCUGACAGUGCGGGCUUUUUUUUUCgaccaaaggu] |
| *aroH* | Transcriptional | Forward | [anti-anti-terminator,1786218,1786293,-65,guggauuaacAGUACCAAUUAUUCGGUAGAAGAGAUUGCCACCAAGAUCCUCGAUAUCAUGGGCCUuagucgccga] [anti-terminator,1786257,1786314,-71,caccaagaucCUCGAUAUCAUGGGCCUUAGUCGCCGAAUGUACUAGAGaacuagugca] [terminator,1786279,1786342,-118,ggccuuagucGCCGAAUGUACUAGAGAACUAGUGCAUUAGCUUAUUUUUUUGUUaucaugcuaa] |
| *ivbL* | Transcriptional | Reverse | [terminator,3851128,3851175,-124,gcgacugacgAAACCUCGCUCCGGCGGGGUUUUUUGUUaucugcaauu] [anti-terminator,3851146,3851228,-1825,guauacuuucAGUGUUGACAUAAUACAGUGUGCUUUGCGGUUACCAGCCGCAGGCGACUGACGAAACCUCGCUccggcggggu] [anti-anti-terminator,3851179,3851251,-124,auugcgcguuCUGCGCGGAACACGUAUACUUUCAGUGUUGACAUAAUACAGUGUGCUUUGCGGuuaccagccg] |
| *aroA* | Transcriptional | Forward | [anti-anti-terminator,957902,957977,-123,ccgcuggaagGCGUUAAAGCGCUGACAGACUUCAUGGUUGAGUUCGAACGCCGUCACGGUUAAUGCcgaaauuuug] [anti-terminator,957941,958002,-46,gaguucgaacGCCGUCACGGUUAAUGCCGAAAUUUUGCUUAAUCCCCACAGCcagccugugg] [terminator,957974,958023,-135,uuugcuuaauCCCCACAGCCAGCCUGUGGGGUUUUUAUUUcuguuguaga] |
| *ldcC* | Transcriptional | Forward | [terminator,209589,209636,-166,aguucugaaaAAGGGUCACUUCGGUGGCCCUUUUUUAUcgccacgguu] |
| *cysB* | Transcriptional | Forward | [terminator,1331688,1331737,-194,uuuuauaaacAAAGGGUCGCGAAAGCGGCCCUUUUUUAUUgcauauuauu] |
| *dcyD* | Transcriptional | Reverse | [terminator,1997551,1997602,-14,uaaugacaaaAAAGGGCGCUUUCACUAGCGCCUUUUUUAUUUacgcguuuuc] [anti-terminator,1997574,1997664,-2012,auggcacucuGCAAGCCCUUUCCGAAAAAUGGUUUGGUGCUGAUGUGACCAAAUAAUCAGCAUAAUGACAAAAAAGGGCGCuuucacuagc] [anti-anti-terminator,1997614,1997672,-104,gcaaaaagauGGCACUCUGCAAGCCCUUUCCGAAAAAUGGUUUGGUGCUgaugugacca] |
| *pheP* | Transcriptional | Forward | [anti-anti-terminator,600927,600990,-189,gauucuguggGGAACGGGGGCUGGUUCAGAGGUGAUGAGCCGGAUUGCCGCGCCgaugauuggc] [anti-terminator,600955,601014,-115,gaggugaugaGCCGGAUUGCCGCGCCGAUGAUUGGCGGCAUGAUCACCGCaccuuugcug] [terminator,600978,601036,-84,gccgaugauuGGCGGCAUGAUCACCGCACCUUUGCUGUCGCUGUUUAUUaucccggcgg] |
| *argF* | Transcriptional | Reverse | [terminator,289644,289708,-145,ucgauccaauGUCUUUCUGCUUCUGCAGAGAAUCGGAGGCAGAUACGAUUAUUUUcacacacgga] [anti-terminator,289670,289703,-59,ccaaugucuuUCUGCUUCUGCAGAgaaucggagg] [anti-anti-terminator,289675,289729,-54,gacucgaccuGGUUGUAGAAUUCGAUCCAAUGUCUUUCUGCUUCUgcagagaauc] |
| *proY* | Transcriptional | Forward | [anti-anti-terminator,420080,420143,-171,ggccauuaucUGGGAUCGCGCGGCAGGUCGUCAGGUGACCUCCAGCGCUCACUAaaucacugaa] [anti-terminator,420112,420176,-617,aggugaccucCAGCGCUCACUAAAUCACUGAACAUUUGUUUUAACCACGGGGCUGcgaugccccg] [terminator,420144,420198,-225,cauuuguuuuAACCACGGGGCUGCGAUGCCCCGUGGUUUUUUAUUguguugaugg] |
| *alaA* | Translational | Forward | [terminator,2405526,2405563,-6,uucuaagcugACUUCCACGGCAGGGAGUggcgauaaca] |
| *alaC* | Translational | Reverse | [terminator,2496322,2496373,-151,ccaucguggcAGCGUCGCUCGGACGGUCCGGGCGCUAACGUUaaucugagga] |
| *metF* | Transcriptional | Forward | [anti-anti-terminator,4130345,4130441,-189,ugaaauauauUGAAUUAUCAUAGGAUUAGGCCGGAUUAAGCGUUUACGACGAAUCCGGCAAGAAGCAAUAAGUACAUGGUUAGUUUAuauuugcagu] [anti-terminator,4130396,4130463,-68,aauccggcaaGAAGCAAUAAGUACAUGGUUAGUUUAUAUUUGCAGUCCGGUUUGCUUUgcauaccgga] [terminator,4130429,4130486,-134,uuauauuugcAGUCCGGUUUGCUUUGCAUACCGGAUUUUCUUUUUCUUaccauccuga] |
| *sstT* | Transcriptional | Forward | [anti-anti-terminator,3237660,3237743,-2269,uuuucgcaccGAUGCUGGCCUGUUCCCCUCACCCUAACCCUCUCCCCAAACGGGGCGAGGGGACUGACCGAGUCcuuuuuugau] [anti-terminator,3237718,3237775,-84,ggggacugacCGAGUCCUUUUUUGAUGUUGUCAUCAGUCUGGAAGCCGcacguuggcu] [terminator,3237749,3237795,-65,caucagucugGAAGCCGCACGUUGGCUUUAUUUUUAUgucaaagaaa] |
